# Advances in chitosan-functionalized textile biomaterials: properties, modification strategies, performance evaluation and multifunctional applications

**DOI:** 10.1039/d6ra01244b

**Published:** 2026-08-13

**Authors:** Md. Hanif Munshi, Md. Fahmid Islam, Md. Hafizul Islam, Nikhilesh Bhowmick, Tauqir Khan, Md. Kamruzzaman, Tarikul Islam

**Affiliations:** a Department of Applied Chemistry and Chemical Engineering, Faculty of Engineering, Gopalganj Science and Technology University Gopalganj 8105 Bangladesh mkamruzzamandu81@gmail.com; b Department of Textile Engineering, Uttara University Dhaka 1230 Bangladesh; c Department of Textiles, Merchandising, and Interiors, University of Georgia Athens Georgia 30602 USA tarikul@uga.edu; d Department of Textile Engineering, Jashore University of Science and Technology Jashore 7408 Bangladesh

## Abstract

Chitosan (CS), a naturally derived biopolymer found in chitin, has emerged as a promising candidate for enhancing textile biomaterials due to its biocompatibility, biodegradability, and intrinsic antimicrobial properties. In recent years, considerable attention has been given to the functionalization of textiles with CS, aiming to improve performance characteristics while enabling new applications across biomedical and industrial domains. This review highlights current advancements in CS-coated and CS-based textile composites, focusing on innovative fabrication techniques and chemical modification strategies that improve material properties such as mechanical strength, moisture regulation, bioactivity, and environmental sustainability. Functionalization approaches, including surface grafting, crosslinking, and nanoparticle incorporation, have significantly broadened the scope of CS in textile engineering. The resulting biomaterials exhibit multifunctionality, making them suitable for wound healing dressings, drug delivery platforms, fire retardant and antimicrobial clothing. Furthermore, the convergence of CS with smart textile technologies has enabled the development of intelligent materials responsive to environmental stimuli. This review also explores the challenges in scalability, durability, and regulatory approval that currently limit the commercialization of CS-based textiles. By integrating material science insights with application-driven perspectives, this paper provides a comprehensive overview of how CS is reshaping the future of functional textiles. The review ends by identifying gaps in research and proposing future directions for sustainable innovation and interdisciplinary collaboration.

## Abstract 2

Chitosan-coated textile composites enhance mechanical strength, biocompatibility, and environmental sustainability, enabling multifunctional performance for biomedical, industrial, and smart textile applications.

## Graphical abstract

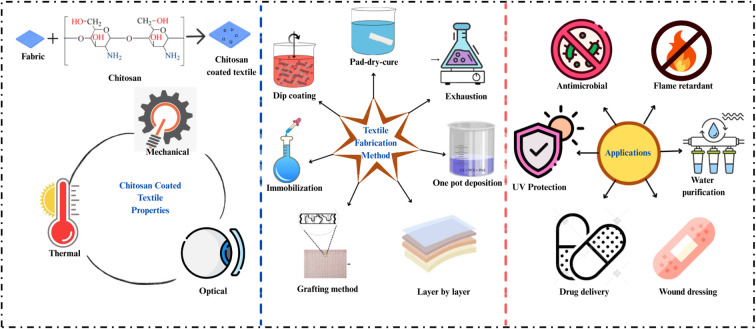

## 1. Introduction

Biomaterials are attracting a lot of attention due to their biocompatibility, biodegradability and multifunctional performance in biomedical and textile applications. Among them, chitosan (CS) functionalized textile biomaterials are very promising due to their antimicrobial activity as well as versatile functional properties.^1,2^ CS is a copolymer composed of *N*-acetyl glucosamine and glucosamine, consequential from chitin, a biopolymer found in various organisms such as the exoskeletons of crustaceans, mollusks, algae, insects, and fungi cell walls.^3,4^ CS has the reactive OH and –NH_2_ groups, serving as a flexible raw material for producing a wide variety of goods with improved qualities such as polyelectrolyte, antimicrobial, antioxidant, biocompatibility, gel-forming, easy processability, and metal chelating, with application in food, pharmaceutical, medical, and agriculture.^5,6^ Chitin is insoluble in typical solvents, but when deacetylated to produce CS, it shows superior solubility and significant biocompatibility and degradability. While the solubility of CS is significantly improved, and it has flexible functionality, it still has some demerits, such as low functional effectiveness and limited solubility. The above problems can be overcome by modifying the structure to enhance its properties.^3^ For functionalizing the CS structure, key chemical modifications are the *O*-substitution, *N*-substitution, and cross-linking with other compounds, which improve the overall physical and chemical properties.^7^

CS shows a favorable range of values for main properties, including surface tension, viscosity, adhesion strength, and mechanical performance. Native or modified CS can be applied as a permanent coating, finishing, or sizing agent that significantly protects yarns during weaving.^8^ For the amine functionality, the CS can again be modified with various reagents to insert tailored properties. Cross-linking modification is needed to enhance washing fastness and improve moisture retention when adjusting the fabric with the CS derivative. The withdrawal of heavy metals and dyes with CS-grafted fabrics enhances dyeability.^9^ Besides the functional benefit, CS can impart biological, antiviral, and anti-odor properties to textiles, particularly in the vital parts of textile items such as sportswear, underwear, shoes, and socks. Fabrics tailored with CS and CS nanoparticles (CNPs) have the best antimicrobial qualities against both Gram-positive and Gram-negative bacteria, as well as fungi and yeasts, as compared to untreated fabrics, which enable them to be used in combination with antimicrobial drugs in the treatment of acute skin injuries.^10,11^ CS also has an antistatic effect in polyester (PET) fabric when Sol–gel coatings include silica sol/CS formulations.^12^ These have been made from a biomass-based, sustainable coating designed to biomaterials are attracting a lot of attention owing to their biocompatibility, biodegradability and multifunctional performance cotton fabrics with substantial fire retardancy by a dip-pad-dry-cure process. In this process, phosphorylated CS acts as an intumescent fire retardant, which is anchored into the fiber surface by the combination of chemical grafting and electrostatic interaction.^13,14^ CS functionalized viscose fibers have stronger strength than raw viscose fiber; the formation of hydrogen bonds with CS is the main reason for the strength used in the biomedical sector.^15^ Carboxymethyl chitosan (CMC) enhances antibacterial activity by adding carboxymethyl and quaternary ammonium groups to CS against *Escherichia coli* (*E. coli*) and *Micrococcus luteus* (*M. luteus*).^12^ Trimethyl CS ammonium, a water-soluble cationic derivative formed *via* quaternization with methyl iodide and NaOH, shows reduced molecular weight (MW) and remains soluble across pH levels above 25% quaternization. These derivatives offer good flocculating and antistatic properties. CS hydrogels, grafted with lactic and glycolic acid without catalysts, improve water interaction and are biocompatible and biodegradable, usable for wound healing and drug delivery.^8^

The dyeing of cotton and viscose fabrics, as well as pre-treated fabric with CNPs and CS, was tested for rubbing, washing, and sweat fastness. The findings show that all of the dyes previously mentioned range from acceptable to outstanding, and salt-free dyeing is also acceptable.^16,17^ CS/silk fibroin composites promote cell function, tissue repair, and antibacterial properties by acting as slow-release carriers for substances such as hydroxyapatite, graphene, silver, and magnesium.^18^ CS-based hydrogels are hydrophilic polymer biomaterials characterized by a three-dimensional cross-linked network. CS hydrogels can be used in drug delivery due to their ability to regulate drug release through pH and temperature (temp) -responsive methods, and they can also serve as carriers within the network. CS and its derivatives make a vital contribution to the healing phase of wounds. The wound healing process mainly includes four parts: inflammation, hemostasis, skin remodeling, and proliferation.^19^ This hydrogel can also provide a moist environment, absorb wound exudate, and accelerate healing.^20^ The CS-based self-healing hydrogel dressing also focuses on promising results for wound healing.^21^ Additionally, their swelling ability, porosity, and mechanical stability make the hydrogels effective candidates for use as a scaffold for tissue engineering. Beyond these applications, CS hydrogels are used in protein purification and dye separation from wastewater.^22^ Chemical modification of CS with melamine and sodium pyrophosphate as nitrogen and phosphorous sources, and glyoxal worked as a crosslinking agent.^23^ Treated cotton fabrics showed enhanced flame-retardant properties. Furthermore, metallic NPs can be integrated into CS fibers to produce medical textiles. These textiles are manufactured for therapeutic or diagnostic purposes, including biosensors and drug systems. CS microfibers, made by wet spinning, are strong and stretchy, with impressive tensile strength (TS) and modulus. CS grafted polyethylene glycol fibers outperform pure CS fibers, with 50% higher TS and 200% better modulus. These fibers also have temp-regulating properties, making them ideal for smart textiles.^20^ CS is one of the multifunctional biopolymers that has captured notable attention owing to its tunable optical properties, inherent bioactivity, and film-forming capability with high adhesion strength.^24^

Despite some beneficial accounts of CS, its derivatives, and NPs, there are limitations associated with its widespread use. Although CS is approved for application in dietary supplements, wound dressing, and cartilage formulations, there has not yet been a single formulation of CS-based drug approved for mass-marketing reported so far.^25^ Poor thermal stability (THS), and inadequate mechanical strength are also some of the major limitations of CS.^26^ CS is derived from crustaceans, and crustacean allergy has become a pressing global challenge that affects public health.^27^ So, people sensitive to crustaceans might show allergic reactions when CS is incorporated in textiles. This review explores recent progress in CS-functionalized textile biomaterials, highlighting key performance improvements, commonly used modification and functionalization approaches, and their expanding applications across multiple sectors. By bringing together current research findings, ongoing challenges, and future opportunities, this paper aims to serve as a clear and practical resource for researchers, academics, and industry professionals working toward the development of sustainable, high-performance textile materials for next-generation applications.

## 2. Functionality of CS

**Fig. 1:**
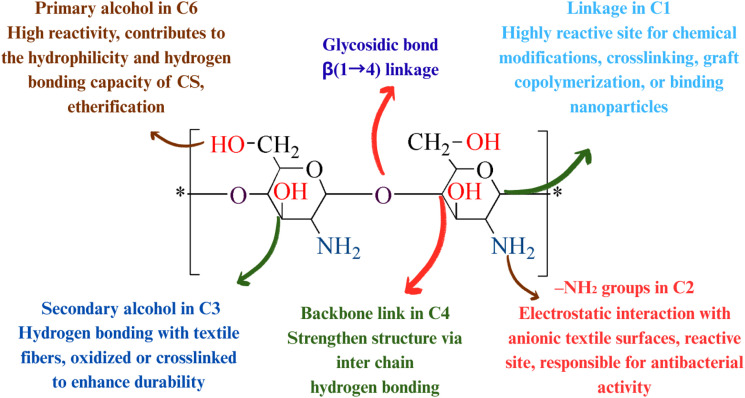
Functionality of CS structure (created with Canva).

CS can be categorized as a copolymer composed generally of 2-acetamide-2-deoxy-β-d-glucopyranose and 2-amino-2-deoxy-β-d-glucopyranose (glucosamine) units (*N*-acetylglucosamine) linked by glycosidic β (1 → 4) bonds, which have many good sides, such as biocompatibility, biodegradability, accessibility, and no toxicity, showing vital antibacterial potential^28–30^(see Fig. 1).

There are many crosslinking agents for functionalizing CS, such as 1,2,3,4-butanetetracarboxylic acid and maleic acid (MA).^31^ Through the crosslinking agent, the covalent bonds can occur between the OH groups and –NH_2_ groups of CS of cotton cellulose.^32,33^ The presence of hydroxyl group at C_6_ is primary alcohol whether at C_3_ is secondary alcohol. These hydroxyl groups are highly reactive and contribute to the hydrogen bonding through the crosslink to enhance the durability. On the other hand, the existence of –NH_2_ groups at C_2_ position, taking part in electrostatic interaction with the surfaces of other phenomena. –NH_2_ group is the most identical group of CS as well as reactive site. Moreover, backbone link in C_4_, enhancing the strengthen structure through the inter chain.^31^

## 3. Properties of modified CS

### 3.1 Mechanical properties

Mechanical properties are altered by the chemical or structural nature of the polymer, but are moreover determined by the spinning conditions.^34^ Mechanical properties include TS, elongation at break (EB), stiffness, hardness *etc.* which can be enhanced by the chemical modification of CS. The TS of the fabric depends on many factors, such as fabric structure, yarn twist, and yarn count *etc.*^35^ CS modified cotton fabric showed enhancement for both the TS (warp & weft) and the EB, due to cross-linkage produced between oxidized CS and cotton fiber, alternating the weaker binding sites of fibers, preventing the fiber breakdown, which stress assembly occurred, thereby improving the strength of the fiber.^36,37^ Therefore, oxidized CS treatment enhances the strength of the altered fabrics.^35^ The functionalization of PES fabric with homogenized CS gel by the pad-dry-cure process resulted in excellent antimicrobial efficacy and improved mechanical and spectral properties of the fabric.^38^ However, CS functionalization with cellulose chains can also yield reduced mechanical strength.^39,40^ The presence of silver nanoparticles (AgNPs) in cotton-CMC composites, as reinforcing nanofiller, improves the mechanical properties owing to strong interfacial interactions that enhance stress transfer and structural integrity. In addition, they are known for their antimicrobial activity by releasing Ag^+^ ions which damage microbial membranes and cellular functions thus resulting in cell death. The dual function of the composites makes them promising for biomedical and textile applications.^41^

### 3.2 Thermal properties

The THS is the property ability of a material to retain its physical characteristics when exposed to high temp and is typically evaluated by measuring the weight loss during heating. CS, 2-acetamido-2-deoxy-β-d-glucose unit linked *via* a β-(1–4) bonds, shows lower THS.^42^ Generally, CS shows thermal degradation at 250–300 °C whereas modified CS with crosslinked with citric acid changes the decomposition to 250–300 °C temp through inducing intermolecular hydrogen bonding. At increasing temp, the composite (CS–ZnO) coated with cotton fabric exhibited better THS than the commercial CS.^43^ The initial weight loss was due to water evaporation. In contrast, the second and more significant weight loss was linked to the degradation of leading chains and the formation of volatile compounds. There is a weight loss after generating the heat.^44^ During degradation, Phytic Acid (PA) and CS favor the development of a thermally stable char layer, which acts as a barrier to block the heat and thereby slows down the oxygen transfer and the second step weight loss step, indicating higher THS.^45^

### 3.3 Optical properties

Optical characteristics of the biopolymers CS and chitin have been evaluated for a wavelength range of 250 to 750 nm.^46^ Multifunctional CS/AuNPs coatings showed attractive optical properties, UV-light protection.^47^ Dyed and printed cotton fabrics altered with nano chitosan (NCS) and polyurethane-based finishing showed improved Ultraviolet Protection Factor (UPF) efficiency that defines how effectively the material can block the UV radiation, with treated samples showing higher UPF levels than untreated samples.^48^ Functionalize CS contributes to increased UV protection, UPF improvement in fabrics, and enhances color strength, K/S value.^37,49^ Modified CS with coated fabric shows optical properties due to the aromatic or conjugated structure in CS, chromophores, and metal complexes. It can be used in smart textiles, sensors, and UV protection clothing, among other applications. Here, we summarize the various CS modification techniques, their reactive sites, and their effect on fabric characteristics. It shows enhancement of antimicrobial activity, mechanical strength, dye uptake, UV shielding, and durability in various textiles (see Table 1).

**Table 1:** Mechanical, thermal, and optical properties of modified CS^a^

Modified CS	Reactive sites of CS	Properties	Additional value	Characteristics	Textile	Ref.
NMA-HDCC	Formation of covalent linkages between NMA, HDCC, and OH groups of cellulose	Whiteness values, (78.1–67.1)	85.3% bacterial reduction (30 cycles)	Increase the antibacterial properties, dye uptake, fixation percentage, K/S values, and dye fastness of reactive dyes	Cotton	50
		K/S value: (4–9)				
CS-based waterborne polyurethane	–OH group of cellulose and –NH_2_ group of CS	Tear strength: (20.7–24.4) g L^−1^ for warp (12.1–20.2) g L^−1^ for weft; TS: (550.41–625.12) g L^−1^ for warp, (232.76–302.23) g L^−1^ for weft	After 3 washings	↑TS, tear strength, ↑antimicrobial activity, and ↓EB	PES/cotton	49
CS coated PES	N/A	BF at (CV%): (2.978–11.765) %	Mean water contact angles (WCAm): 0.00–43.51	Multifunctional premise and favor ecological effects, tensile properties, and K/S values	PES	37
		EB (CV%): (2.451–11.689) %				
		TS: (27–58) MPa				
		Thermal characteristics: (2.0–4.0)				
		K/S value: 1.51–3.27				
CS grafted-β-cyclodextrin	–NH_2_ group of CS and the glutaraldehyde	↓TS: From 12.8 to 6.8	Hydrophilicity: (12–22)	Sustainable, ↑efficacy of keratinase, ↑storage ability &THS, ↓TS.	Wool	51
		Temp (60–75 °C) & (75–90 °C); grafting yield of β-CD (70–100) %, (100–80) %				
CNPs	–OH group of cellulose and –NH_2_ group of CS	N/A	Antibacterial against *Staphylococcus aureus* (*S. aureus*) & *E. coli* retained after 5 & 1 washing	↑Antibacterial, ↑positive charge density of CNPs	Viscose	52
CS functionalization into PES/cotton blend	CHO group attaches with cellulose, and the opposite end forms intramolecular bonds with CS for –NH_2_ group	BF: (726.50–864.00) N for cotton, (996.00–1225.67) N for PES/cotton blend	After 5 washings, there is a lower quantity of CS	↑Stability, antibacterial, lower amount of CS after washing	PES/cotton blend	53
CS functionalization of cellulosic	–OH group of cellulose and –NH_2_ group of CS	BF: (364.5–850.0) N for cotton, (829.2–1212.0) N for PES/cotton blend	Loss of durability after 10 washings	Antimicrobial, ↓durability, mechanical properties, whiteness, and yellowing	Cotton and cotton/PES blended	31
		EB: (14.90–20.88) %				
N, O-CMC	–COOH and aldehyde groups provide linking sites for –NH_2_ groups of CS	Strain rate: 150 mm min^−1^	After 5 washings, CS ↓from 1.13 to 0.48	TOCN, used to enhance viscose with CS, ↑antibacterial, and mechanical properties	Viscos, cotton fibers	40
		BF: ↓11% in warp,2.5% in the weft				
CS/AuNPs	–NH_2_ groups of CS	Temp_peaks_ of 1st derivative (°C), Soy: 295.1	Washing fastness up to five laundry cycles	↑Antibacterial, THS, optical properties, UV-light protection	Soybean protein-based knitted rib	47
		Soy + CS: 352.9				
		Soy + AuNPs: 292.3				
		Soy + CS + AuNPs: 424.1				
		Reflectance: ≈34%				
CS-genipin	–COOH groups of anionic cyclodextrin	After thermal treatment, (30–70) % chlorhexidine release	Remains effective after washing	Release chlorhexidine, long-lasting antibacterial efficacy, cytocompatibility	PES nonwoven	54
	Polymer and –NH_2_ groups of CS					
CS-modified cotton thread	–NH_2_ group of CS	↑Wicking rate is almost 8 cm after being treated for 15 min	↑Hydrophilicity after storage for 3 months	↑Wicking rate, ↑colorimetric signal intensity, stable wettability	Cotton thread	55
PA/CS polyelectrolyte complex for wool	–OH and –NH_2_ groups of CS	TS: (274.4–276.8) N	After 10 washing cycles	↑Flame retardancy, lower cost, good washing durability	Wool	45
		Flexural rigidity: 1.131–1.61	Whiteness index: 71.6–81.8			
		Highest thermal degradation state: (250–450 °C)				
CS-AgNPs	Positively charged group of CS polysaccharides	Highest thermal degradation state:(300–400 °C)	For CS-AgNPs, antioxidant: 96.08 & antibacterial: ≥ 98.0	Sustainable option to chemical functional agents	Linen	56
		K/S: 2.8				
NCS coated cotton fiber (NCCF)	–NH_2_ group of CS	↓In weight observed within temp of 243°C-360 °C	For NCCF, adsorption volume (Mmol g^−1^): Cd(ii): 4.76C; r(VI): 12.5; Pb(ii): 6.40	Removing heavy metals, a greater initial adsorption rate, and adsorption capacities	Cotton	57
CNPs	–NH_2_ and –OH group of CS	For CS NPs	After 10 cycles, retained antimicrobial activity	↓Fabric strength, wrinkle recovery, EB, enhance stiffness and sustainable	Cotton fiber	58
		TS: (330–356.2)				
		EB (%): 9.23–9.75				
		At 500 °C, the weight loss of 81.77% for CS and 55.38% for CNPs				
Salicyl-imine-CS	–NH_2_ group of CS and carbonyl group (C=O) of the salicylaldehyde (SA)	TS:37.4 N mm^−2^, 32.8 N mm^−2^, 40.8 N mm^−2^	Average swelling capacity: (120–280) %	Swelling rate capacity, dehydration rate, burn wound healing, and antimicrobial	Cotton gauze	59
			Dehydration rate: (15–75) %			
CS hydrogel-coated cellulosic	–NH_2_ group of CS	After washing, TS (warp, weft)	AP after washing	Antimicrobial, ↑hydrogel-bound rate, ↓AP, ↑wettability, tensile behavior and the comfort properties	Cotton	60
		Non-activated: 475N, 510N	Anionic: 33,9 L m^−2^ s^−1^			
		Cationized: 525N, 550N	Cationic: 39,3 L m^−2^ s^−1^			
		Anionized: 550N, 540N	Untreated: 39,4 L m^−2^ s^−1^			
CS in citric acid functionalization onto cotton	–NH_2_ group of CS with –OH group of cotton	TS: (672–811) N	Antibacterial activity 70–89%	Wrinkle-free, fire retardant, UV shielding, ↓TS, ↑bending distance, ↑crease upturn & whiteness index	Cotton	61
			Antioxidant activity (93.4–94.73) %			
CMC	–NH_2_ groups of CMC	TS: (14–20) MPa	Water absorbability: 240–280%	Antimicrobial, ↓safety risk, and low environmental impact impact	Cotton	62
		Flexibility: 13.9 mm, 14.1 mm	Washing cycle: 50			
CS-coated woven cotton	–OH, group of cotton, acid groups of photonic nanosphere bond with –NH_2_ groups of CS	Thermal transition: (300.8–434.6 °C), highest degradation temp: 350 °C	ΔH (J g-): 30.8–148.2	↑Degradation temp, ↓enthalpy, high washing durability, light fastness, and cost efficiency	Woven cotton	63
		K/S: 51.23–616.9				
CS-bound cellulosic	–OH, group of cotton and –NH_2_ group of CS	Major decomposition: (300–450 °C)	Wash fastness: 4–5	Excellent durability, antibacterial activity (>76%)	Cotton	32
			K/S: (0.799–1.005)			
CS, chitooligosaccharide	–NH_2_ group in CS and quinone group in dopamine quinone	BF: (710–770) N for warp, (300–310) N for weft	After 20 washing cycles, antibacterial percentage between 83–100 by deposition, (85–100) % by padding	↑Bonding fastness & UV protection, antibacterial rates >96%, well mechanical abrasion	Cotton	64
Short-chain oxidized CS	–COOH group of CS and –OH on cotton cellulose	110 MPa stress for cotton textile, 100 MPa for oxidized CS-cotton textiles	After 30 wash cycles, the inhibition rate was over 98%, 99.99%	Antibacterial, antiviral, durability, TS, ↑slipperiness & drapeability, ↑AP	Cotton	65
CS nanocapsules	–NH_2_ group of CS and sodium tripolyphosphate	Rapid thermal decomposition: (300–380 °C, 72.6% mass loss)	Encapsulation ability: 93.18%, loading ability: 14.54%	↑Release rates, electrostatic adsorption, slow-release properties, THS	Cotton	66
CS hydrogel composite	–NH_2_ and –OH group into the silk	Highest stress 68 MPa, at 15% strain rate	K/S value: 1–21% after 20 washing cycles, retain 70% antioxidant activity	↑Adsorption, ↑controllable release of luteolin, ↑UV protection, mechanical, strong antioxidant	Silk	67
		Highest thermal degradation, *T*_max_: 337.2 °C				
Quaternized CS Schiff base-TiO_2_-ZnO nanocomposites	–OH group of fabric and –NH_2_ group of CS	Thermally stable up to 245.98 °C, for NC1, up to 226.28 °C for NC_2_	After 10 washing cycles, antibacterial activity for NC1 (TiO_2_-rich): (12–20), for NC_2_ (ZnO-rich): (23–31)	Strong, sustainable antimicrobial, higher UV protection, homogeneous distribution, higher depositing density	Cotton	68
CS/TiO_2_ functionalized polypropylene	Aldehyde groups in glutaraldehyde and –NH_2_ groups in CS	N/A	Contact angle: 49.4–142.3 °C	↑Hydrophilicity, photocatalytic properties, ↑antimicrobial activity	Polypropylene nonwoven	69
CS-based formulations on cellulose-based linen	–COOH acid groups of citric acid and –OH groups of cellulose, –NH_2_ group of CS	TS: (387.2–774.1) N	Absorbency: (2.50–2.80)	Wrinkle-free, antibacterial, flame-retardant, UV protective	Linen	70
		Residue at 480 °C: weight loss (22.40–42.68) %	After 20 washes, antioxidant activity: 50.85			

a BF: breaking force; AP: air permeability; LOI: limiting oxygen index; NMA-HDCC: *O*-acryl amidomethyl-*N*-[(2-hydroxy-3-dimethyl-dodecylammonium) propyl] CS chloride

Table 1 summarizes various CS-modified textile materials, highlighting their reactive sites, resulting properties, and textile types. CS, known for its –NH_2_ and –OH functional groups, readily forms covalent linkages with cellulose, PES, wool, silk, linen, and other fibers, producing multifunctional textiles. Across the studies, CS modifications significantly enhanced hydrophilicity, antimicrobial activity, and mechanical strength, while also improving THS, dyeability, and UV protection.^66^ For instance, CS-coated cotton fabrics showed marked increases in flame retardancy and rheological properties, while NMA-HDCC-treated cotton exhibited higher antibacterial activity and dye fixation.^50,71^ Similarly, CS-based polyurethane composites improved tear and TS, and CNPs or CS-AgNPs imparted sustained antioxidant and antibacterial effects even after multiple washing cycles.^49,52,56^

Moreover, the incorporation of nanocomposites like TiO_2_, AgNPs, and expandable graphite introduced flame retardant, photocatalytic, and antioxidant properties, broadening CS application scope.^56^ Functionalization often involved reactions between the –NH_2_ group of CS and –OH, –COOH, or aldehyde groups of the textile substrates, yielding durable bonds that maintained efficacy over several washes. CS-linked systems (*e.g.*, CS-genipin, CS hydrogel, or CS/β-cyclodextrin) enhance controlled release, wound-healing potential, and biocompatibility.^54^ CS-modified textiles demonstrate sustainability, biodegradability, and multifunctionality, making them promising alternatives to conventional chemical finishes. These modifications not only improved mechanical and comfort-related features (*e.g.*, wicking rate, wettability, air permeability) but also introduced eco-friendly flame retardancy, wrinkle resistance, and UV shielding, establishing CS as a versatile and green bio-based finishing agent for advanced textile applications.

## 4. Methods of CS functionalization and textile coating

This contains different types of CS used in textile substrates, reaction conditions (temp, catalyst, concentration, and time), and bonding mechanisms, all of which are covered in functionalization and textile coating. Table 2 outlines different methods of CS functionalization (pad dry, LBL, dip coating, grafting, exhaustive, one pot and immobilization) with the fabrication factors (time, concentration, temp, *etc.*) with several textile fabrics. CS, with different Degree of Deacetylation (DDA), MW, and viscosity, affects the overall properties, interaction, and factors are extracted from difference sources and that was fabricated in different types of textile fabrics.

**Table 2:** Different CS modification methods applied to textile substrates along with reaction conditions and interaction mechanisms^a^

Methods	Types of CS	Textile	Factors (reaction mechanism)				Interaction/bonding	Ref.
			Temp.	Catalyst/modems	Conc.	Time		
Pad-dry-cure	MW: 80	Cotton and cotton/PES blended fabrics	100 °C and 150 °C	MA and 1,2,3,4- BTCA	25 g L^−1^ MA, BTCA, 10 g L^−1^ SHP, and 2 g L^−1^ NF-9	5 min	Cross-linking	31
	DDA: 90							
	CS from crab shells	Viscose, cotton	Room	2,2,6,6- TEMPO	0.5% CH	30 min	Irreversible binding of CS	40
	MW: low							
	Quaternized CS	Cotton	60 °C	Schiff base-TiO_2_–ZnO nanocomposites	N/A	15 min	Cross-linking	68
	N/A	PES/cellulosic blended	150 °C	Curcumin	N/A	1 min	Cross-functional linkage	72
	Viscosity: 159 mPa	Woven fabric	100 °C	TiO_2_ + ZnO	0.2%	3 min	Embedding	73
	DDA: 95%			NPs				
	DDA: 90%, viscosity: 1000 cPs	Poly (ethylene terephthalate)	N/A	Amano lipase A	3 g L^−1^	110 °C for 2 min dry	Hydrolysis is carried out by a batch-wise method	74
	MW: 150 KDa							
	*N*,*N*,*N*-trimethyl CS	Cotton	Room	TPP	1:50 ratio	30 min	Cross-linking	75
	MW: 82 000							
	DDA: 77.4%							
	CS polyelectrolyte complex	Wool	70 °C	Citric acid	2% (w/v)	10 min	Cross-linking	45
	DDA: 90%							
	Freeze dryer CNPs	Cotton	85 °C	KMnO_4_	N/A	3 min dry	Cross-linking	58
	DDA: 89.2%, MW: 2.4 × 10^4^							
	Salicyl-imine-CS biopolymer	Cotton gauze	Room	SA	1% CS	1 hour	Cationization	76
	MW: medium							
	MW: 15 KDa, DDA: >75%	Cotton	130 °C	N/A	1%	1 min	Adsorption	77
	CNPs	Cellulose fabric	150 °C	Acetic acid	30 g L^−1^	3 min	Cross-linking	78
	MW: 2.4 × 10^4^ Da, DDA: 89.2%	Cotton and viscose						
	MW: low (50–190 kDa)	Cotton and polyamide 6,6	95–150 °C	Glacial acetic acid solution	2% CS	3 and 3 min	Cross-linking	79
	CNPs viscosity: 1860 cP; deacetylation degree: 79%	Cotton	40–100 °C	Tripolyphosphate (0.001 g/100 mL) with CS (0.2 g/100 mL)	0.2 g/100 mL	30 min	Ionotropic gelation method	80
	CS-based recipe	Linen	100 °C and 140 °C	Citric acid	1% CH	Dry and cured 3–5 min	Cross-linking	81
	MW: medium	Cotton and wool	85 °C	Acetic acid	0.5% w/v	5 min	N/A	82
	DDA: 75–85%							
	CMC	Cotton	180 °C	AgNPs	CMC (0.1 wt%)	Immerse 30 min	Esterification or amidation	83
	Mw, 15 kDa; DDA: [95%; substitute degree, 80%]							
	Poly-imidazolium vanillyl-grafted CS (oligochitosan)	Cotton	70 °C	6% Aqueous acetic acid	3% CH	3 min	H bond	84
	Golden and silver-golden CS hydrogels	Cotton	65–70 °C	1% Acetic acid	2%	30 min	Covalent	85
Layer-by-layer (LbL)	CS polyelectrolyte complex (CTS, mw 150 kDa	Wool	N/A	Dopamine quinone and the –NH_2_ group	5 g L^−1^	5 min	Schiff base reaction	64
					CTS, COS			
					ε-PL			
	MW: low viscosity: 20–300, DDA: 80% ± 5%	Nonwoven	25 °C	Genipin	0.5% (w/v)	15 min	Cross linking	54
	Phosphorylated CS	Polyamide 66	N/A	PAS	1% CS	30 min	Grafting and cross-linking	86
	DDA: 80–95%							
	Viscosity: 50–800 mPa.s							
	MW: 150 000; DDA: 90.1%, nitrogen content: 7.2%	Cotton	110 °C	Citric acid and sodium lignin sulphonate with boric acid	1%	3 min	LbL	61
Dip coating	N/A	Viscose nonwoven	Room	1.0% CH_3_COOH	0.5%	24 h	Cross-linking	87
	DDA: 95%	Cotton	50 °C	Glycidyl methacrylate	N/A	10 min	N/A	88
	Mw = 350 kDa; DDA: 85%	Cotton gauze	70 °C	1% of acetic acid	0.06–0.50, wt%	30 min	H bond	89
	CS nanofiber aqueous gel (2.5% w/v, 85%)	Cotton	Room	Silver phosphate NPs	1 wt%	20 min	Immobilizing	90
	Deacetylation ≥ and 90%, density ≥ 0.6 g mL^−1^)	Superhydrophobic cotton	Pre-dried at 80 °C	Poly-dimethyl siloxane	N/A	Baked for 2 min at 160°	Cross-linking	91
						C		
	DDA: 80.0–95.0%; viscosity:50−800 mPa·s)	Cotton	N/A	N/A	1gm	12 h	H bond	92
	NCS	Cotton	70 °C	Melamine phosphate	N/A	10 min	N/A	93
	MW: low 50 kDa, DDA: >75%, viscosity 20 cps)							
	N/A	Silk	N/A	2% acetic acid	2% CH	15 min	H bond	67
Grafting	Source: shrimp shell	Polypropylene	50 °C	Tannic acid and post-modification	3.75 g of CS dispersed in 250 mL of acetic acid	4 h stirrer	Cross-linking	94
	DDA: 87%							
	MW: 50−190 kDa, DDA: 75–85%	Wool	50 °C	Laccase/2,2,6,6-TEMPO mediated oxidation	30 mL, 0.4%	6 h	Cross-linking	95
Spray coating method	MW: 132 KDa; DDA	Nonwoven PES	80 °C	N/A	10 mg mL^−1^	40 min	N/A	96
	85%							
	MW: 190–375 kDa	Cotton	37 °C	Inorganic ZnO	50 µg mL^−1^	Overnight	N/A	97
	DDA: ≥75% and viscosity: >200 cps							
Exhaustion method	Immobilization of CS	Soybean protein-based knitted rib fabric	70 °C	Au NPs	2%	30 min at 40 rpm	N/A	47
	CS (viscosity 1860 cps, DDA: 79.0%), Penta sodium TPP	Cotton and viscose	40, 60, and 80 °C	N/A	3, 6%	2 °C/min	N/A	98
	MW: 45 KDa	PLA-knitted	70 °C	N/A	1% CH	60 min	N/A	99
	DA: 1%							
One pot method	CS polysaccharide	Linen	90 °C	Ag NPs	N/A	60 min	N/A	56
	CMC	Cotton	25 °C	NaBH_4_ solution	1% CS	30 min	Coordination bond	100
	MW: 15 KDa, DDA: >95%							
Immobilization	CS microspheres	Cotton	60 °C	Acetic acid	N/A	24 h dry	Cross-linking and covalent	101
	DDA: 85%							

a TEMPO: tetramethylpiperidine-1-oxyl; SA: salicylaldehyde, TPP: tripolyphosphate; PLA: polylactic acid; MP: melamine phosphate; PVA: polyvinyl alcohol; PAS: polyacrylate sodium; SHP: sodium hypophosphite; BTCA: 1,2,3,4-butanetetracarboxylic acid

Across the studies, CS modifications with various methods significantly enhanced hydrophilicity, antimicrobial activity, and mechanical strength.^66^ One of the most essential methods is pad-dry-cure to fabricate CS functionalized textiles such as cotton, wool, cotton/PES blend, viscose, *etc.*, with various interactions (crosslinking, H-bond). Among all methods pad-dry-cure is the most widely used and industrially practical technique. It is particularly effective for cotton, viscose, wool and cotton PES blends due to good crosslinking between CS amino groups and cellulose hydroxyl groups.^31,45^

Similarly, the layer-by-layer and dip coating methods are used to fabricate textiles like cotton, polyamide, wool, cotton/PES blend, viscose, *etc.*, functionalized CS with various interactions (crosslinking, H-bond, grafting, *etc.*).^86,102^ Some of the important CS modified textile fabrication methods, such as pad dry, LBL, simple coating method, grafting, exhaustion, and one pot deposition method are discussed in the following sections. These fabrications not only improved mechanical and comfort-related features but also introduced eco-friendly flame retardancy, wrinkle resistance, and UV shielding, establishing CS as a versatile and green bio-based finishing agent for advanced textile applications.

### 4.1 Pad dry method

The textile finishing process is typically conducted in a continuous manner where chemicals or finishes are applied to fabric processed through padding, followed by drying and curing. In textile wet processing, pad-dyeing is a conventional and frequently used technique that requires significant amounts of water, chemicals, and energy.^103^

**Fig. 2:**
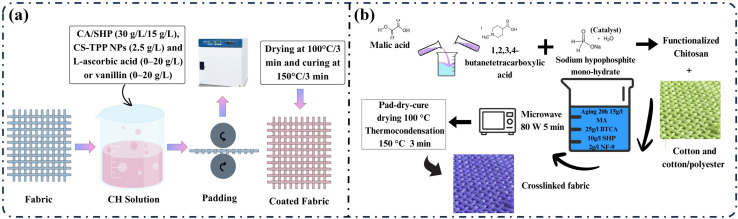
Functionalized CS cross-linking on (a) cellulose and (b) cotton/PES blended fabrics using the pad-dry cure method (created with Canva).

Flinčec Grgac *et al.* (2023) examine that CS was functionalized using malic acid (MA) and BTCA as crosslinking and CS activating agents, with sodium hypophosphite monohydrate serving as the catalyst. This functionalized CS was cross-linked with cotton and cotton/PES blended fabrics. The fabrics were aged in a solution containing CS, MA, or BTCA, SHP, and NF-9. After ageing, they were treated using a microwave. The pad-dry-cure technique was applied by drying at 100 °C for 2 min and thermocondensation at 150 °C for 3 min^31^ (see Fig. 2(b)). A study led by Ibrahim *et al.* (2024) explained that functionalized CS cross-linked with cellulose fabrics in the pad-dry-cure method, followed by drying at 100 °C for 3 min and curing at 150 °C for 3 min.^78^ Cotton and viscose fabric samples were treated with CS-TPP NPs. The CA/SHP study by Refaee *et al.* (2022) showed that quaternized CS cross-linked with cotton fabric by pad dry technology^68^ (see Fig. 2(a)). Initially, the impurities that adhere to the surface of cotton fabric during textile processing are removed by washing with a nonionic detergent. Then, the samples were rinsed with deionized H_2_O and dried. Suspensions of the free ligand and its nanocomposites were assembled by dispersing individual materials with deionized H_2_O, followed by sonication. Later, the dry fabric samples were immersed in the respective suspension and exposed to ultrasonic irradiation. The treated fabrics were then squeezed 100% using the pad dry technology under continuous pressure and cured. Schiff base-TiO_2_-ZnO nanocomposites were used in their study for UV protection and antimicrobial purposes.

### 4.2 LBL method

**Fig. 3:**
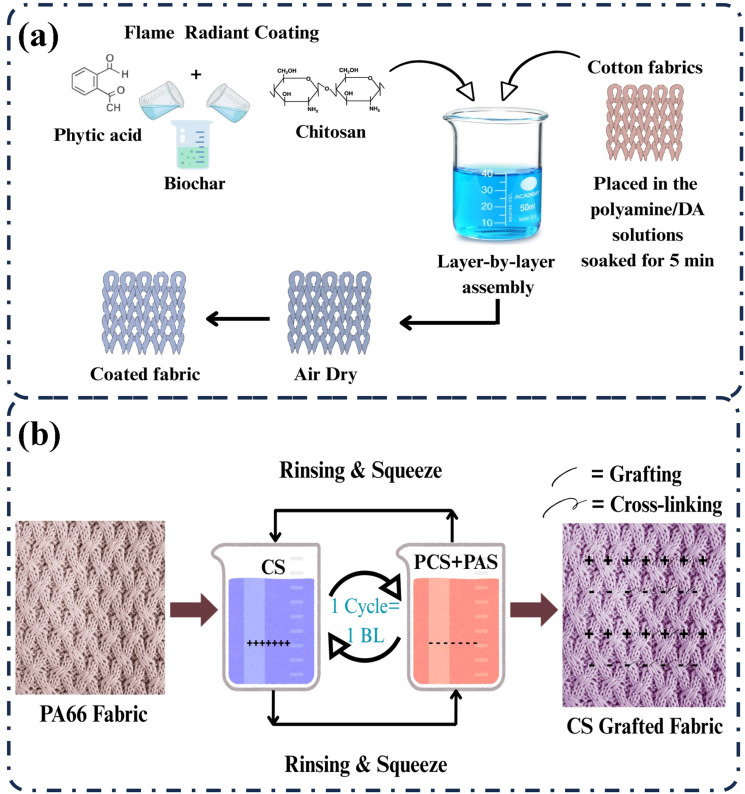
LBL coating of (a) cotton (created with Canva) and (b) PA66 with functionalized CS. Reproduced with permission^86^ Copyright 2020, Elsevier B.V.

Covalent LBL assembly is an influential technique for fabricating functional ultra-thin films that permit nanoscopic structural precision, flexible design, and componential diversity. This approach provides precise control over the surface and interface of materials, allowing for the formulation of a composite where the interfacial and bulk properties of the material can be independently managed.^104^ A study by Cheng *et al.* (2022) found that A fire-retardant coating composed of PA, CS, and biochar (BC) was assembled by the LBL assembly. They showed that CS blended with cotton fabric, a Schiff base reaction. The cotton fabrics were immersed in the polyamine/DA solutions and then pressed *via* a padder. Finally, the fabrics were air-dried naturally. In this stage, the A/Q cross-linkage reaction occurred rapidly on the surface of the cotton fabric^64^ (see Fig. 3(a)). Safi *et al.* (2020) examined the synthesis of functionalized CS through the LBL method. 1% CS solution was prepared with citric acid and SHP. On the other hand, an aqueous solution containing BA and SLS was formulated. Cotton fabric was prepared by padding with CS and dried. The fabric was again padded with SLS. The sample consisted of one bilayer of CS and SLS and cured.^61^ Also, Kundu *et al.* (2020) reported the coating of polyamide 66 (PA66) fabric with functionalized CS using the layer-by-layer (LBL) method. The fabric was immersed in the PCS solution for repeated deposition cycles, with controlled immersion and rinsing steps. They observed that grafting and cross-linking interactions formed between functionalized CS and the fabric surface, leading to stable coating formation. The adsorption and rinsing steps were carefully controlled, and no drying was performed between successive deposition cycles, as shown in Fig. 3b.^86^

### 4.3 Dip coating/simple coating method

**Fig. 4:**
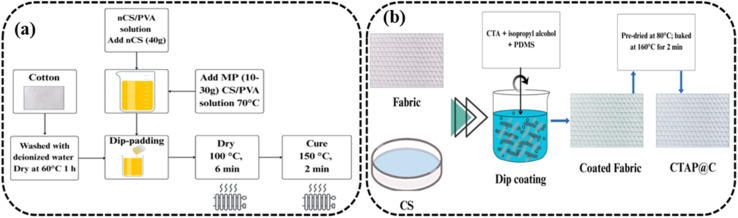
To achieve superhydrophobic property, cotton textiles are modified using (a) NCS *via* dip-padding and (b) with dense CS *via* dip-coating (created with Canva).

Deposit coatings onto a wide range of substrates, including ceramic, metallic, polymeric films, and fibrous materials.^105^ The process can be described as applying aqueous-based liquid phase coating solutions onto the surface of a substrate.^106^ In general, the target materials are submerged in solutions, which are then directly deposited onto the substrate surface. As a result, the wet coating is subsequently evaporated to form a dry film.^107^ Tan *et al.* (2024) proposed that high-density CS was cross-linked with superhydrophobic cotton fabric using the dip-coating method. The untreated fabric was washed and dried for subsequent use. A cotton sample was then immersed in a mechanically mixed solution containing CTA, prepolymer of Polydimethylsiloxane (PDMS), isopropyl alcohol, and curing agent of PDMS. The cotton fabric was thoroughly impregnated and continuously stirred to ensure uniform distribution. To prepare antibacterial cotton fabric, the fabric was immersed and rolled to achieve a liquid carrying rate of 85%, pre-dried, and baked^91^ (see Fig. 4(b)). A study by Makhlouf *et al.* (2022) showed that NCS blended with cotton fabric treated by dip-padding technology. Firstly, the fabric was washed with deionized water and then dried, for coatings carrying NCS/MP, the NCS solution was incorporated into the PVA solution. Then, the addition of MP to the PVA/NCS solution to formulate NCS coatings. Cotton fabric samples were immersed in these coatings and had 100% pick-up within three padding cycles. The coated fabrics were dried at 100 °C for 6 min and cured at 150 °C for 2 min.^93^ Superhydrophobic cotton, achieved through NCS dip-padding and dense CS dip-coating, is shown in this method (see Fig. 4(a)).

### 4.4 Grafting method

**Fig. 5:**
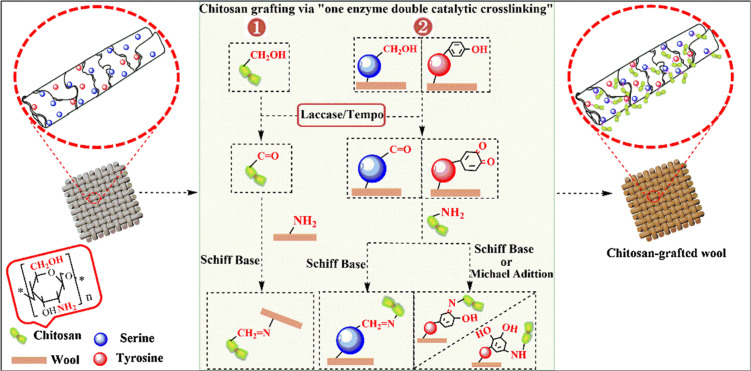
Functionalized CS modified wool fabric by the CS Grafting method. Reproduced with permission^108^ Copyright 2020, Elsevier B.V.

Functionalizing textiles *via* grafting modification has appeared as a favorable strategy, presenting a vital prospect for the textile industry. Grafting modification is a universal process that incorporates covalently connecting functional molecules or polymers to the surfaces of textile fibers or fabrics.^47^ A study conducted by Fouilloux and his collaborators demonstrated that functionalized CS was cross-linked with polypropylene using tannic acid and post-modified through quaternary ammonium by the grafting method. The cross-linking reaction utilized tannic acid, a bio-sourced and biocompatible cross-linker, under mild conditions in an aqueous solution. Due to the steric hindrance of TA, the cross-linking rate remains low but effective, leaving a portion of the primary amines of CS accessible for post-modification by quaternary ammonium grafting using method.^94^ In another study by Wang *et al.* (2021), wool fabric was modified with functionalized CS through CS grafting using a one-enzyme double catalysis method. The wool fabric was treated with a prepared solution. The CS solution was combined with a laccase, TEMPO, L-ser, and PHAD. This mixture reacted with oxygen. This reaction product was precipitated in ethanol to obtain the final product^108^ (see Fig. 5).

### 4.5 Exhaustion method

**Fig. 6:**
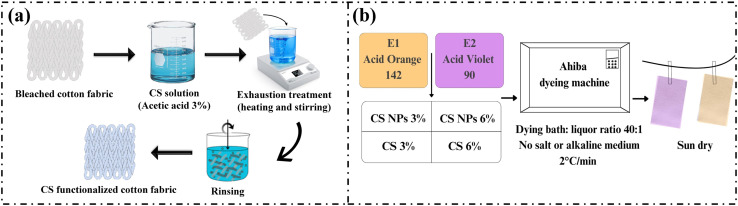
Employing the exhaustion method, functionalized CS were combined with (a) scoured and bleached cotton fabric; and (b) CS/CNPs were blended with cotton and viscose textiles (created with Canva).

Commercial microcapsules were applied onto the fabric surface by either padding or bath exhaustion. During the finishing process, resin was used as a binder. Therefore, thermal treatment of hot air was carried out to cure the resin and to increase adhesion of the microcapsules to the fabric.^109^ The exhaustion method is one of the earliest and most commonly used techniques for functionalizing cotton with CS. In a groundbreaking study, John A. Rippon (1984) applied CS to scoured and bleached cotton fabric by soaking the fabric in an acidic CS solution at 40 °C. CS uptake increased with treatment time, temperature, and concentration, showing a strong attraction between CS and cellulose. The addition of sodium chloride had little effect on CS adsorption, suggesting that electrostatic interactions were not the main method of adsorption. Instead, the sorption process mainly relied on van der Waals forces and hydrogen bonding due to the structural similarities between cellulose and CS. At higher CS levels, the exhaustion method achieved deposition similar to pad application while providing a more even distribution of CS on the cotton fibers. This study laid the groundwork for future exhaustion-based CS functionalization of cotton textiles^110^ (see Fig. 6(a)). Another study by Mohamed and his collaborators demonstrated that two viable acid dyes, Acid Violet 90 (E2), and Acid Orange 142 (E1), were applied to cotton and viscose fabrics altered with CS and CNPs to enhance dye absorption and antibacterial properties. The coating of cotton and viscose fabrics with CNPs and CS at different temp in an Ahiba dyeing machine, without using salt or alkaline medium. Finally, the temp was increased to the fixation point at a rate of 2 °C per minute. Then, dyed cotton and viscose fabrics were dried in the open air^98^ (see Fig. 6(b)).

### 4.6 One-pot deposition

**Fig. 7:**
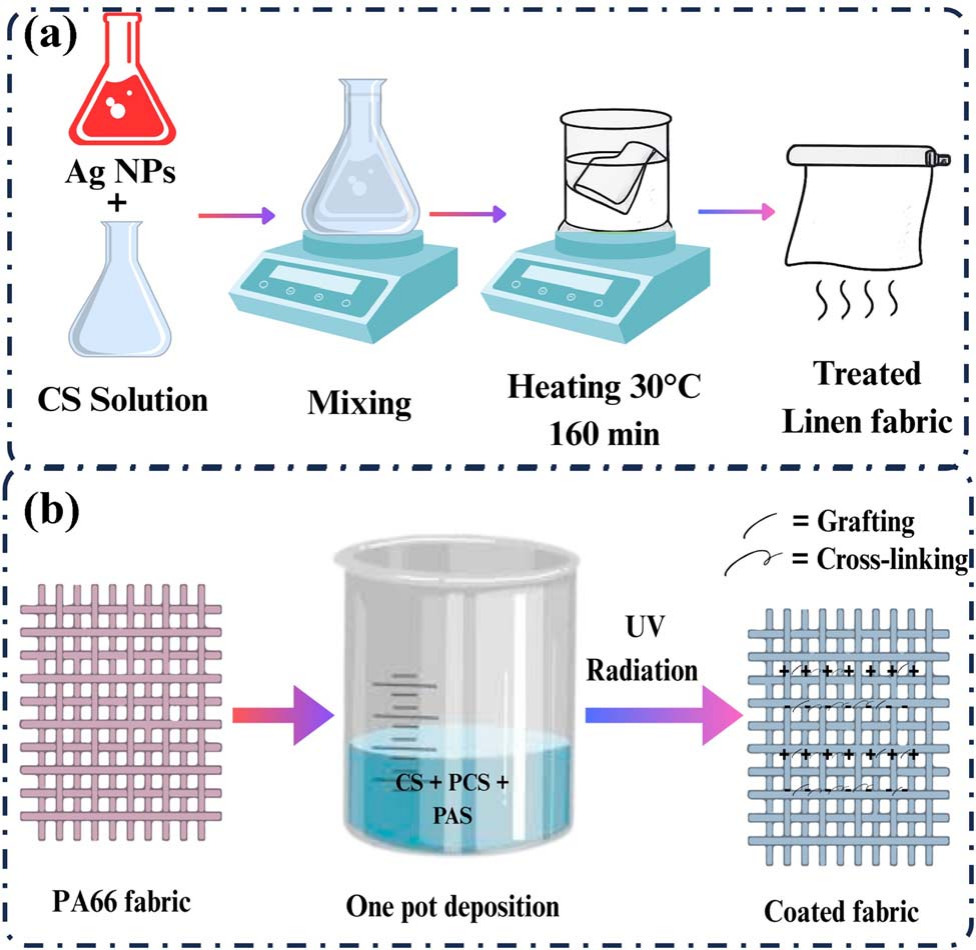
One-pot deposition method applied to blend (a) linen (created with Canva) and (b) PA66 fabrics with functionalized CS. Reproduced with permission^86^ Copyright 2020, Elsevier B.V.

One-pot deposition is a textile fabrication method usually used for multi-functional coating of several textile fabrics Chen *et al.*, 2023). Using gentle polyelectrolytes such as polyphosphate sodium salt and branched polyethylenimine (PEI), a ‘one-pot deposition system’ was developed to create stable polyelectrolyte complex suspensions that imparted flame-retardant properties to cotton fabrics.^86^ A study by Butola *et al.* (2019) examined how using CS polysaccharides can transfer the coloring, antibacterial, and radical scavenging activity of AgNPs to the linen fabric surface through a one-pot deposition method. An AgNO_3_ aqueous solution was developed in distilled water using a conical flask and then added to the CS solution. Subsequently, linen fabric was submerged in the colloidal AgNO_3_ and CS. Afterwards, pineapple extract was added dropwise to the CS-Ag solution. To improve coloration on the linen fabric, the mixture was heated. Finally, the fabric was thoroughly rinsed and dried in the shade at room temp^56^ (see Fig. 7(a)). Kundu *et al.* (2020) reported a one-pot deposition technique for the application of CS-based flame-retardant coatings on PA66 textiles. CS, phosphorylated CS, and sodium phytate were mixed in a single bath at pH 5.5 and the fabric was immersed for 5–10 min by this method. Then the treated fabric was exposed to UV irradiation to promote the grafting and cross-linking between the coating components and the PA66 fibers, to improve the coating durability and flame-retardant performance. The one-pot deposition approach is a more simple, rapid and scalable strategy for functionalization of textiles with CS-based coatings compared to conventional layer-by-layer assembly^86^ (see Fig. 7(b)).

### 4.7 Immobilization method

**Fig. 8:**
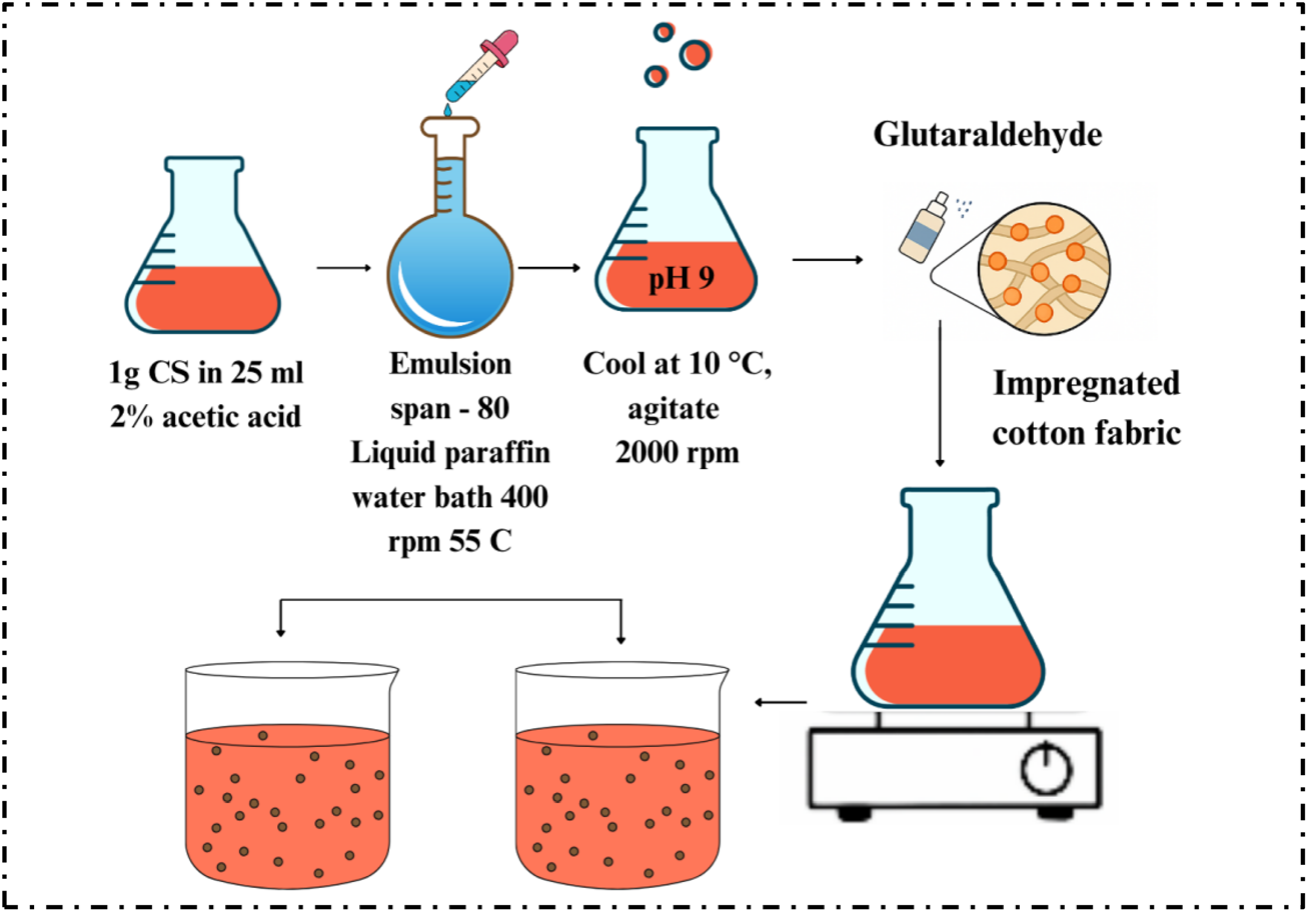
Cotton fabric with CS lysozyme microspheres by immobilization (created with Canva).

In textile coating, an immobilization method refers to fixing functional molecules, like CS, onto the fiber surface so that they do not easily wash away.^111^ Cerón *et al.* (2023) demonstrated the saturation of cotton fabric with CS lysozyme microspheres through covalent attachment, conducted at 40 °C for 30 minutes with a stirring speed of 40 RPM. A bath solution that accumulates CS and microspheres (100 mg per 1 g of fabric) was employed, with a bath ratio of 1:40 (cotton fabric: bath). To prepare CS, acetic acid was dissolved and stirred for 24 h. The resulting gel was filtered with methanol. An emulsion consisting of Span-80 and liquid paraffin was prepared while stirring in a water bath. CS gel was mixed, and the emulsion was stirred until fully homogenized. After homogenization, the emulsion was cooled in an ultra-thermostatic bath. The pH was maintained, and glutaraldehyde was slowly added *via* spray system, and the reaction was maintained^101^ (see Fig. 8).

## 5. Performance and application areas of modified CS

**Fig. 9:**
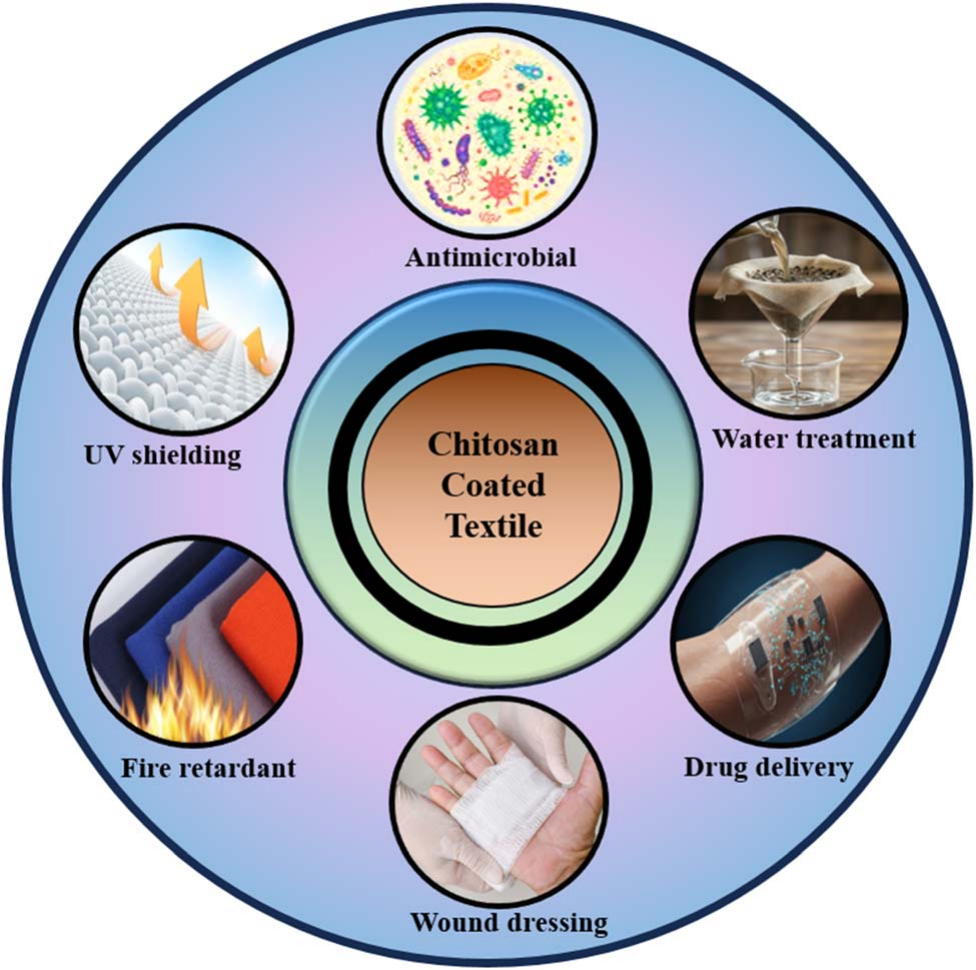
Applications of CS-coated textiles in antimicrobial, UV-protective, flame-retardant, water treatment, drug delivery and wound dressing areas (created with Canva).

CS and its chemically modified derivatives exhibit enhanced performance in terms of solubility, antimicrobial activity, biocompatibility, and functional stability compared to native CS. Structural changes like as grafting, crosslinking, and Schiff base production considerably improve mechanical strength, moisture retention, and bioactivity. The main uses of modified CS in textiles, including antimicrobial finishes, burning retardancy, ultraviolet (UV) ray protection, water purification, drug delivery, and wound dressings, are shown in Fig. 9, along with the specific CS types and crosslinking employed in each.

Greater antimicrobial and antifungal efficacy, higher LOI/THS, higher UPF, pollutant removal, controlled drug release, and improved wound healing properties are just a few of the valuable advantages it highlights. Real-world capability is shown by durability following removal and quantitative test results (zones of inhibition, UPF/LOI values, swelling, encapsulation efficiency, *etc.*) Overall, the table shows how CS can improve textile safety, protection, and biomedical functions in a sustainable and multipurpose way (see Table 3 and 4).

**Table 3:** Application of CS coated textile in different fields

Application area	Textile type	CS type/crosslink	Application	Valuable data	Ref.
Antimicrobial properties	Cotton & PES	CS polysaccharide	Antimicrobial modification of textile materials	↓Flexibility, ↓viability, ↑growth of bacteria, antibacterial 65% after 25 washing	112
	Cotton 100%	Medium MW, CNPs	Enhance dyeing properties and antimicrobial activity of cotton	↑Nitrogen content, ↑antimicrobial activity, ↑temp, decomposition, ↑ K/S values ↑cationic site, ↑yellowness, Gram-positive bacteria exhibit higher efficiency than Gram-negative bacteria	80
	Cotton	CS/ZnO nanocomposite, MW: 3800–20000 Da	Antibacterial performance onto cotton textile and applicability in dye degradation	Tested *versus S. aureus* (14 mm ZOI), *Bacillus subtilis* (*B. subtilis*) and *E. coli*, ↑photocatalytic activity, ↑separation efficiency of photogenerated electrons and holes on the interface	113
	Pure cotton	CS hydrogel- functionalized	Cotton fabric antibacterial activity, pH-sensitivity, and physical characteristics	Tested against *L. monocytogene*, *E. coli*, *S. aureus*, improved antibacterial activity, highest inhibition zones for *S. aureus* and *L. mono*, ↑hydrophilicity, ↑moisture management, ↑thickness, ↓TS	114
	Cotton woven	CS- and aloe vera	Thermal comfort characteristics of altered cotton woven textile	↑GSM & thickness due to strong cotton affinity, ↑absorbency, ↓with *A. vera* concentration, ↓TS with ↑finishing agent, ↓THS	115
	PES/cotton	N/A	CS in cotton and PES/Cotton blend	↑Zeta potential, ↑shrinkage and swelling ↑yarn count and strength	53
	100% cotton	Low MW, CNPs	Enhancement of dyeing and antimicrobial ability of cellulosic textile	Bacteria incorporate *S. aureus*,*B. subtilis*, *E. coli*, and proteus, fungal strains are *Aspergillus niger*, ↑mechanical, thermal, and optical properties of cotton	116
	Cotton	Low MW, CS/Ag/clay nanocomposite	Durable multi-functional properties	Well dispersed in Cs matrix, ↑TS of cotton, ↑THS, complete bacterial inhibition and ↑antifungal activity	117
	Cotton fibers	ZnO-CS/pandan leaves	Antimicrobial potential of advancing antimicrobial textile technology	Tested against *B. subtilis*, ↑performance, ↓corrosion properties, 23 mm inhibition zone, ↑mechanical strength	118
	Superhydrophobic cotton	CS and TiO_2_ NPs	Coated superhydrophobic cotton textile with increased antibacterial and UV-shielding activity	80% enhancement in the UV protecting, ↑contact angle, high level of adsorption of TiO_2_, tested against *E. coli* and *S. aureus* bacteria, bacterial reduction	119
	Linen (epi = 50, ppi = 40, GSM = 143)	CS-AG NPs	Facile synthesis of antibacterial property and free-radical scavenging fabric	Tested against *S. aureus* and *E. coli* functionalized linen exhibit antibacterial limiting of 99.68% (*E. coli*) and 98% (*S. aureus*), antioxidant activity of treated linen increased to 96.8%	56
	Gauze	CMC	Evaluating antifungal agent	↑Polarity and solubility, ↑surface coverage, ↑cell death, ↓crystal unit size	120
	Non-woven 100% cotton (NWC) (45 g m^−2^)	Medium MW [480 000 da]	Non-woven textile wound dressing carry LBL placed hyaluronic acid and CS	↑HA concentration ↓swelling, ↓air and vapor permeability, quaternized fabrics show higher swelling at 6 h, then drop due to increased crosslinking	121
	Knitted	CS/AuNPs	Enhance UV-light protection and antimicrobial feature	↑THS, inhabiting *E. coli* (96.26%) and *S. aureus* adhesion (99.94%), ↑UPF	47
	Viscose	TEMPO-oxidized cellulose nanofibrils/CNPs	Durable antibacterial medical textiles	Tested against *E. coli* & *S. aureus*, ↓bacterial cell growth, ↑washing durability	52
	Cotton/PES	Cu/CS	Enhance antimicrobial feature rich plasticized starch	↑Water absorption, demonstrating *S.aurous*, *E.coli*, ↑stability, ZOI (14 and 6–7), ↑mechanical properties	122
Fire retardant	Graphene-modified	CMCNPs	Green flame retardant	High surface area, ↑THS, ↑conductivity, ↑flammability, ↑electrical conductivity, tested against *E. coli* and *S. aureus*, ZOI (15 and 16 mm) biggest (24 and 28 mm)	123
		MXene/CS	Firefighting, home automation, and smart transportation	↓Heat release rate 66%, excellent flame retardancy, ↑rougher surface, ↑flexibility	95
	Knitted cotton	CS and APP by LBL assembly	Produce fire retardancy on knitted cotton textile	↑Fire-retardant, ↑THS, 16.6 mass% deposition	124
	Viscose	DD = 80–95%	Fire retardancy and antibacterial activity of viscose textile	↑THS, viscose retained 39.0%, LOI value of 29%, 99.99% antibacterial rate, tested against *S. aureus* & *E. coli*	125
	Cotton	CS and phytate	Antibacterial treatment for inflammable natural textiles	↓Smoke and heat production, maintains an irregular shape and rough surface, ↑flame retardancy, ↑char residues, ↑levoglucosan, antibacterial rate 99.83%, tested against *E. coli*	126
	Polyamide 66 textiles	CS, sodium alginate, and metal salts	Flame-retardant coatings in textiles	LOI reached up to 25.5%, peak heat release rate by 34%, ↑char residues, ↑coating stability, ↑thermo-oxidative stability	127
	Cotton (100%)	Mn = 200 000; DD of 85.0%	Textile with promising fire-retardant and antibacterial effect	LOI is 29.7%, antibacterial percentage of *E. coli* and *S. aureus* is 95.1% and 99.9%, ↑AP, ↑residual value, ↑resistance to oxidative degradation	
	Cotton	Mn = 200 000; DD of 85.0%	Sustainable bio-based coatings	Tested against *E. coli* (87.5–95.1%) & *S. aureus* (99.9%), ↑weft breaking force, LOI reached 29.7%, ↑THS	128
	PES/cotton blend (65T/35C, 120 g m^−2^)	CS/PA	Sustainable flame-retardant and dripping-resistant fabrics	↑Flame retardancy, ↑char formation, LOI value of 17.3%, ↑THS	129
	100% cotton	CS and nano-TiO_2_	Fire retardancy of dyed and undyed textiles	↑Blue dyed 5.19%, ↑calico fabric strength 20%, ↑thermal resistance, ↑post-flame combustion performance, ↓thermal decomposition was 17.4%, ↓AP 60.17%	130
	Carbon cloth	Ammonium polyphosphate/CS	Flexible textiles for fire retardancy and electromagnetic interference protecting	AP (69.65 mm s^−1^), water vapor transmittance (273.17 kg·m^−2^·d^−1^), LOI is 36.8%, EMI protecting capacity (59.36 dB)	131
	Pristine cotton		Sustainable flame radiant	LOI 31%, ↓smoke production (63.5–70.0%), high transparency, ↑THS, ↑carbonization of cellulose, ↑thermal oxidative stability	132
	Cotton	CS and nano-TiO_2_	Rapid fabrication of superhydrophobic and flame-retardant coatings	↑THS, ↑weight gain ratefrom 7.5% to 46.0%, ↓hydrophobicity, excellent UV shielding	133
	Cotton	CS/expandable graphite	Diverse fire safety and protection	↑LOI rating from 17% to 43%, functionalized cotton burns inn 760 s where cotton burn within 20 second	134
	Cotton	NCS and PVA, melamine salt of CS phosphate (MCHP)	Fire retardant coatings with improved antibacterial characteristics	PVA/NCS/MP and PVA/MCHP coating ↑LOI of from 17.2% to 57.9% and 58.2%, ZOI of *S. aureus* are 17.3, 26.8 and 30.5 mm, in *E. coli* are 15.5, 24.3 and 27.6, ↑THS, ↓AP	93
	N/A	CS and PA	Durable fire retardant and anti-dripping	LOI value of 34%, after 4 times washing, LOI value was more than 26%, ↓AP	135
	Cotton and PES/cotton	CS and phosphorous acid	Forming of bio-based fire retardants	↑LOI value from 18% to 80.7%, 99.99% antibacterial rate against both *E. coli* and *S. aureus*, ↑THS	71
UV protection	Superhydrophobic cotton	CS and nano-TiO_2_	Increased antibacterial and UV-shielding properties	↑UV protecting properties 80%, antibacterial tested against *S. aureus*, *E. coli* increased to 97.3% and 99.8, ↑water contact angle	119
	PES cotton	NCS and polyurethane	Enhanced UV protection, mechanical and antimicrobial properties	↑TS improved up to 8.1–20%, UPF 10%, tested against *S. aureus* and *E. coli*, ZOI (6.2–9 & 6, 6–9), ↓growth of microbes	48
	Cellulosic fiber	CS and onion skin dye	UV protection and antibacterial treatment	↓Growth of *S. aureus* and *E. coli* percent and 98.03% and 97.20%, UPF value 84.80, 66.17%	136
	Cotton	Nano cellulose and NCS	Sustainable UV protective altered of textile	↓Hydrophilicity, UPF value increase to (14.31–35.29), after laundering cycles 30 (12.55–26.19)	137
	Silk	DD = 80–90%	Increased antimicrobial and UV shielding properties	Inhibitions of 99.08% and 96.71% against *S. aureus* and *E. coli* ↑UPF value from 15–24 to 25–39	138
	PES/viscose	CS/silica NPs	Cu/CS nanocomposites	↑TS by 16%, ↑char yield to 14.8%, UPF was improved by 260%	139
	Bleached cotton		CS and ZnO NPs	UPF was improved from 15 to 40 & against *S. aureus* from 50 to 90 & *E. coli* from 47 to 92	78
	Bifunctional	Silica/CS composite	Bio-microcapsules	93.10% and 99.99% inhibition against and *S. aureus and E. coli*, UPF value of 72.32	140
	Pure cotton	CS/zinc nitrate hexahydrate	Lasting antibacterial, UV-protective and fire retardancy of textile	99.99% & 93.67% antibacterial rates for both *E. coli* & *S. aureus*, less than 5% UV transmittance, ↓smoke release 21.0% and 60.6%	141
	Cotton	CS/melamine and sodium pyrophosphate	Modification of CS-based finishing agent for fire radiant and UV shielding	↓99% of bacterial colonies, ↑polymer crystallinity, ↑THS, ↑LOI value from 18% to 30%, at lowest concentration UPF is 18%, it is increasing during ↑concentration	142
	Silk	Polyaniline/CS	Developed a sustainable flame retardant and UV protection	↑Binding capability, ↑THS, varying UPF value from 30–50, ↓char length, ↑LOI of 28.3%	143
Water purification	Cotton gauze	CMC	Preparation of cotton gauze utilization for water filtration	Roughness decreases gradually, bacterial reduction *E. coli* (95.0) & *S. aureus* (100%)	144
	Cotton gauze	Low viscosity CS, DD 75–85%	Antibacterial water biological filter	Bacterial reduction *E. coli* (95.0) & *S. aureus* (100%), *K. pneumonia* 98%	145
	Cotton gauze	Low viscosity CS, DD 75–80%	Antibacterial water biological filter	Inhabitation with 4 s and 8 s exposure time, ↓pressure drop	146

CS is a vital biopolymer for textile modification because it has multifunctional and eco-friendly properties. It is cationic in nature, which has a strong interaction with negatively charged textile fibers, making it effective for finishing treatments.^147^ One of the major advantages is its antimicrobial property, which has been widely explored for healthcare textiles. Fabricating films and interacting with nanomaterials enhance performance in textiles. For example, medium MW CNPs, when modified with CS, enhance dyeing and antimicrobial properties of cotton.^80^ Moreover, CS improves the hydrophilicity and stability of textiles, which is also beneficial for wearable applications. It shows a vital role in UV protection and has also been incorporated with titanium dioxide or other UV-absorbing agents, making the CS-coated textiles more effective at absorbing UV rays.^119^ Similarly, in flame-retardant textiles, CS contributes to improved THS and reduced smoke release. For example, when knitted fabric functionalized with CS, their fire-retardancy and THS value increased knitted cotton textile.^124^ Finally, in the field of water purification, the active amino and hydroxyl groups in CS provide strong binding sites for dyes and heavy metals.^148^ For instance, CS-modified cotton gauze was prepared for water filtration applications, and it showed a slow decrease in surface roughness and high antibacterial activity toward *E. coli* (95.0%) and *S. aureus* (100%).^144^ The multifunctional performance of CS-coated textiles is highly dependent on the type of substrate, the MW of CS and the nanocomposite formulation. Cotton fabric just predominant in antimicrobial application due to its hydrophilicity and moisture management properties, while superhydrophobic cotton coated with TiO_2_ NPs induces the UV protection almost 80%.^112,119^ For fire retardant textiles, LOI value is reached to over 50% from 17.2% coated with MCHP, NCS and PVA while CS and phytate coated cotton reducing smoke generation with maintaining higher antimicrobial activity ^93,126^. MXene/CS inhibits heat release rate almost 66% with excellent flame retardancy. CS with nano-TiO_2_ shows excellent THS and fabric strength with reducing AP to 60.17%.^95,130^ In UV protective coatings, CS and nano TiO_2_ coated with superhydrophobic cotton exhibits 80% UV protecting properties and higher antimicrobial activity with water contact angle.^119^ CS coated fabric and NCS, NPs shows the UPF value rate more than 35% with hydrophilicity reduction and increasing TS rate.^137–139^ Lastly in water purification system, CS coated cotton gauze shows efficient bacterial reduction and maintaining lower pressure drop.^145,146^ Overall, CS coated cotton type fabric outperformed other substrates with its multifunctional application sectors in antimicrobial, flame retardant, UV protection and water purification. Some of the essential applications of CS modified textiles are studied in the following discussions.

### 5.1. Antimicrobial application

CS, a naturally derived polysaccharide, is an essential material in textile engineering. Its cationic structure and biocompatibility make it helpful in developing smart and sustainable textile finishes. Among its many applications, antimicrobial functionality is the most vital aspect of CS. CS-treated fabrics, especially cotton, have shown significant inhibition against usual pathogens like *E. coli*, *S. aureus*, and *B. subtilis*. For example, the incorporation of CNPs into cotton fabric surfaces shows an increase in antibacterial zones of retardation and improved binding of antimicrobial agents.^80,117^ Butola *et al.* (2019) showed the antibacterial reduction of 99.68% (*E. coli*) and 98% (*S. aureus*), and the antioxidant activity of treated linen increased to 96.8%.^112^ Cotton fabric coated with CS/ZnO nanocomposites showed significant antibacterial property against *S. aureus*, *B. subtilis*, and *E. coli*, and demonstrated superior photocatalytic and dye degradation capability.^113^ ZnO-CNPs synthesized using pandan leaf extract exhibited inhibition zones up to 23 mm and enhanced mechanical strength.^118^ Here, the inhabitation zone is directly connected with contact time; if contact time increases, the inhabitation zone also increases (see Table 3).

**Fig. 10:**
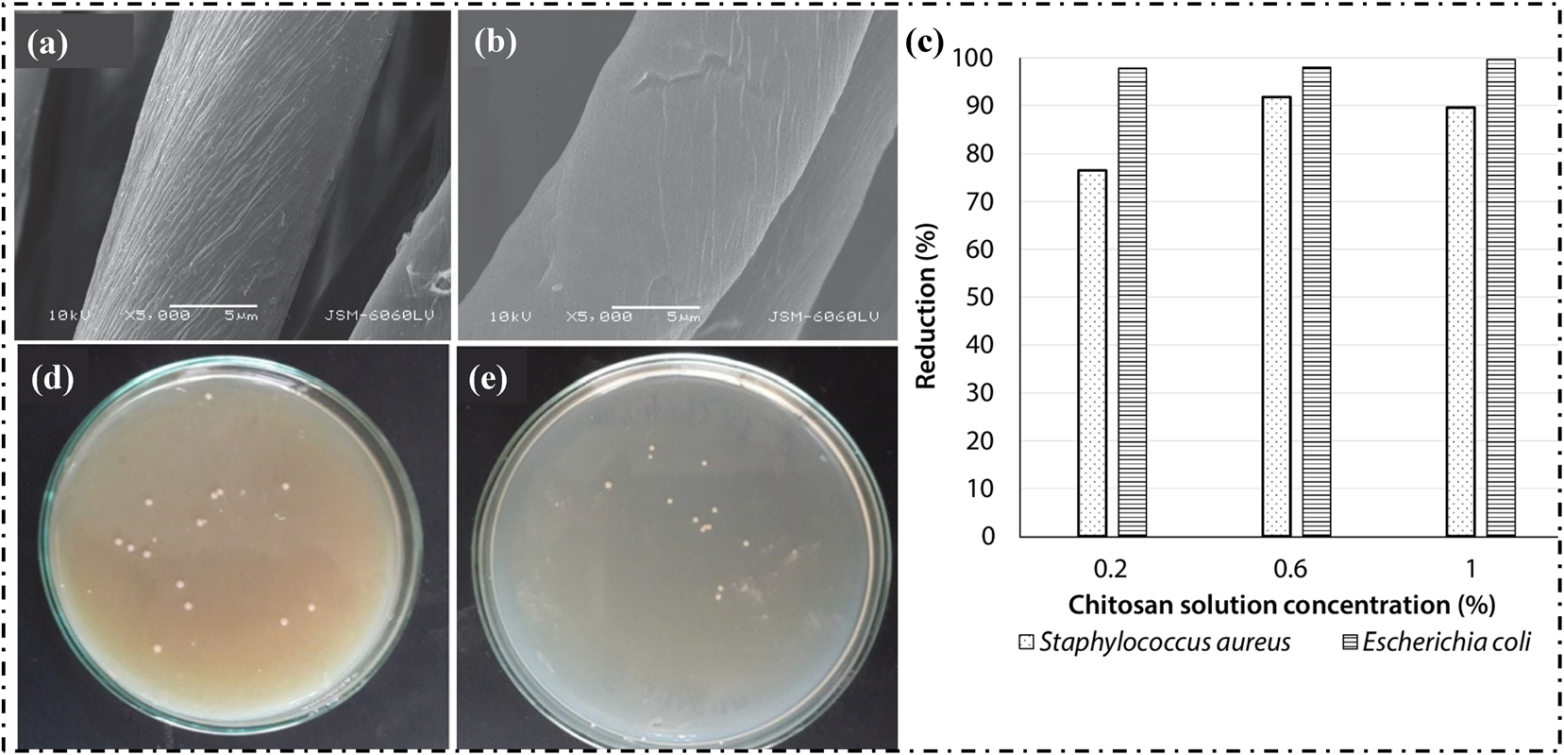
(a) SEM images of cotton yarns; (b) SEM images of cotton with 0.6% concentration of CS solution; (c) reduction % in number of CFU against *S. aureus* and *E. coli* of cotton treated different concentrations of CS. Published under the CC BY license^149^ Copyright 2023, The authors, Tekstilec (d) number of *E. coli* cells after contact with fabric sample treated with 1% CS and (e) number of *S. aureus* cells after contact with fabric sample treated with 1% CS. Reproduced with permission^152^ Copyright 2016, Springer.

According to Toshikj (2023) and Rahman Bhuiyan (2017), treated cotton with different concentration (0.2–1) of CS. Cotton with CS achieved good antimicrobial activity against the bacteria *E. coli* (Gram-negative) and *S. aureus* (Gram-positive). In Fig. 10(a and b), the cotton (scoured and bleached) had clearly expressed fibrils on the surface of the fiber, while after the treatment with CS, the surface became smooth. The absence of agglomerated CS on the surface of CS-treated samples indicates the high quality of the CS solution and the efficacy of the treatment.^149^ Even at a low concentration of 0.2%, the CS-treated cotton in Fig. 10(c) demonstrated over 75% reduction in the number of CFU against both *E. coli* and *S. aureus*, suggesting significant antibacterial action of the CS solution. For *S. aureus*, the decrease rate up to 91.74% at a CS solution concentration of 0.6%. When the concentration of CS solution was raised to 1%, the decrease against *E. coli* reached 99.64%. The accessible amino groups of CS combine with H^+^ to form NH_3_^+^ cations, which subsequently react with the bacterial cell surface negative charge to liberate internal components and disrupt cell functioning.^149,150^ The antimicrobial activity was greater against *E. coli* than against *S. aureus*. Due to variations in the bacterial cell surface, the manner of antibacterial action is a complicated process that varies between Gram-positive and Gram-negative bacteria.^151^ Also, in Fig. 10(d and e), it was discovered that the decrease in bacteria increased when the concentration of CS increased by 1%.^152^

### 5.2. Flame retardancy application

Besides microbial resistance, CS contributes significantly to the improvement of fire retardancy in textiles (see Table 3). When combined with nanomaterials such as expandable graphite, TiO_2_, or MXene, along with phytate, PA, and sodium alginate, this promotes char formation and reduces heat release. This treatment has been applied to many kinds of cotton fabrics, such as knitted cotton, viscose cotton, pristine cotton, and denim. This improves both THS and fire safety. Wang *et al.* (2021) demonstrated that cotton fabrics treated with a CS-MXene combination exhibited reduced heat release rates and increased resistance to combustion. Such treatments present promising directions for safer household and industrial textiles.^95^

**Fig. 11:**
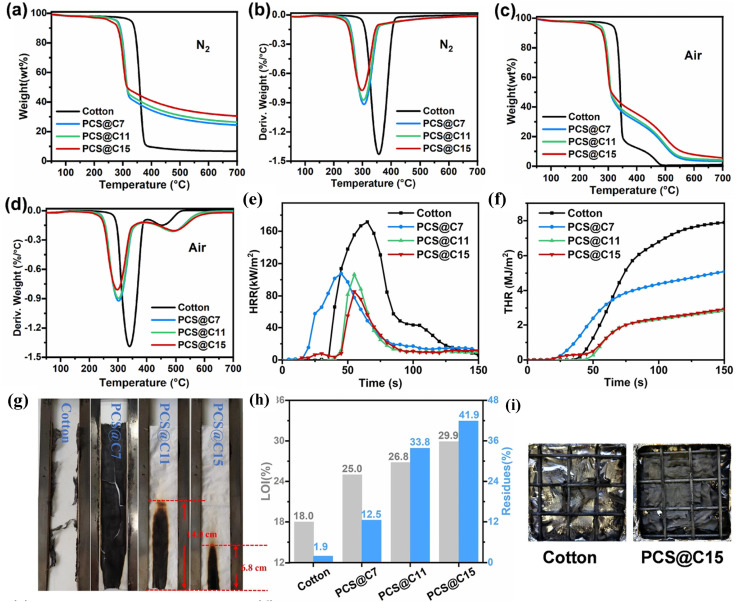
The TGA and DTG curves of cotton and PCS@Cx fabrics under (a and b) N_2_ and (c and d) air atmospheres; (e and f) the HRR and THR curve of cotton and PCS@Cx; (g) vertical burning digital picture; (h) the LOI and Resides of cotton and PCS@Cx and (i) digital photographs of the burning residues. Reproduced with permission^71^ Copyright 2025, Elsevier B.V.

The TGA and DTG curves of PCS@Cx exhibit a consistent thermal degradation trend across all samples under a N_2_ atmosphere, as illustrated in Fig. 11(a and b). The initial decomposition temp (*T*_5%_) of samples in N_2_ and air atmospheres decreased from 327.4 and 313.9 °C for the original cotton fabric to 275.6 °C and 269.7 °C for PCS@C, respectively. The solid residues then underwent additional oxidation and oxygen-induced breakdown, resulting in a secondary weight loss. Every sample in the N_2_ atmosphere showed a one-step breakdown tendency. Phosphite acts as a catalyst in the first stage, promoting the breakdown and carbonization of CS and cellulose. As a result, a stronger carbon layer with improved thermal oxidation stability is created. Significantly, the PCS residue yield increased from 6.8% for cotton fabric to 30.5% for PCS@Cx fabric, a roughly 350% increase. After the cone calorimetry test, the original cotton fabric was nearly entirely burned out with no residue, as seen in Fig. 11(i), whereas the PCS@Cx cloth generally maintained its contour. In the air atmosphere (Fig. 11(c and d), the presence of O_2_ introduced complexity to the decomposition process, resulting in a 2-stage decomposition phenomenon observed across all samples. The initial stage of decomposition mainly involved the processes of dehydration and carbonization, leading to the formation of the residual the subsequent weight loss stage shifts towards higher temp.^71^

The cone calorimetric test (CCT), which provides comprehensive information about flame retardancy, such as pHRR (peak heat release rate), THR (total heat release), and residues, was used to assess the material's combustion behavior under particular conditions (Fig. 11(e and f)). Following PCS coating treatment, the heat release of PCS@Cx was drastically decreased. The cotton pHRR value reduced from 171 kW m^−2^ to 107.7 for PCS@Cx fabric, a 37.2% decrease, and then to 85.3 for PCS@C15, which was more than half of the starting value. The THR value showed a similar pattern, with PCS@C11 and PCS@C15 values declining to 2.9 and 2.8, respectively, indicating a roughly 65% decline. The flame retardancy of PCS@Cx fabrics was investigated through the LOI test and vertical burning test in Fig. 11(g and h). Once ignited, it shows persistent burning until full consumption in vertical burning tests with no significant improvement. Additionally, PCS@Cx materials show good self-extinguishing qualities with limited burning times and damaged lengths during vertical burning tests. Additionally, it was discovered that the LOI value of PCS@C15 increased to 29.9.^71^

### 5.3. UV protection application

UV protection is another critical feature enhanced by CS-based coatings (see Table 3). Fabrics show significant improvement in their ability to deliver UV protection after treatment. Incorporating titanium dioxide, silica, or ZnO NPs with CS increases the capability of the textile to reflect or absorb UV light. Here, they use different kinds of cotton fabrics, but they also use silk and PES as textiles. There is a significant improvement in UPF values after treating cotton with a CS-TiO_2_ system, demonstrating UV resistance of 780% and antibacterial properties of.^119^ This finishing treatment helps hold the fibers in place, preventing them from sticking out of the fabric and forming pills. This multifunctional development supports outdoor wear and fabrics that are both skin-friendly and sustainable.

**Fig. 12:**
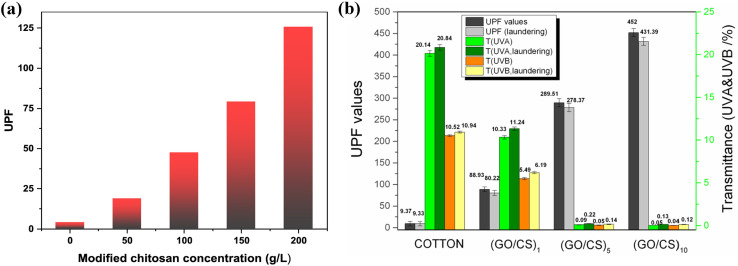
The UPF values and (a) effect of concentration of CS on UV protection. Reproduced with permission^23^ Copyright 2022, Elsevier. (b) UVA & UVB T (%) of cotton and GO/CS deposited fabrics. Reproduced with permission^153^ Copyright 2016, Elsevier B.V.

The values of the UV protection factor (UPF) on cotton and CS treated fabrics are shown in Fig. 12(a). According to data, modified CS-treated fabrics offer superior UV protection compared to untreated cotton fabrics. The UPF value of modified CS-treated fabric at the lowest concentration employed was 18.8, indicating good protection against UV radiation, whereas the average UPF of the untreated materials was 3.8. The percentage and color strength results were consistent with an increasing trend in the concentration of changed CS UPF values.^23^ Tian *et al.* (2016) assessed the UV protection ability of GO/CS deposited fabrics, the UPF values, transmittance of UVA and UVB and the durability after ten times water laundering were measured and illustrated in Fig. 12(b). All of the GO/CS-deposited specimens showed surprisingly higher UPF values than the control cotton fabric, and the UPF values essentially high as the number of GO/CS deposited cycles on the fabric surface increased.^153^

### 5.4. Water purification application

CS-based textile coatings have shown promising potential in water purification applications because of their excellent adsorption capacity and antimicrobial activity. When applied to cotton gauze, CS increases surface polarity and enhances bacterial reduction against strains such as *E. coli* and *S. aureus*.^144^ Its biocompatibility and ability to form stable complexes with pollutants make it suitable for eco-friendly filtration systems (see Table 3).

**Fig. 13:**
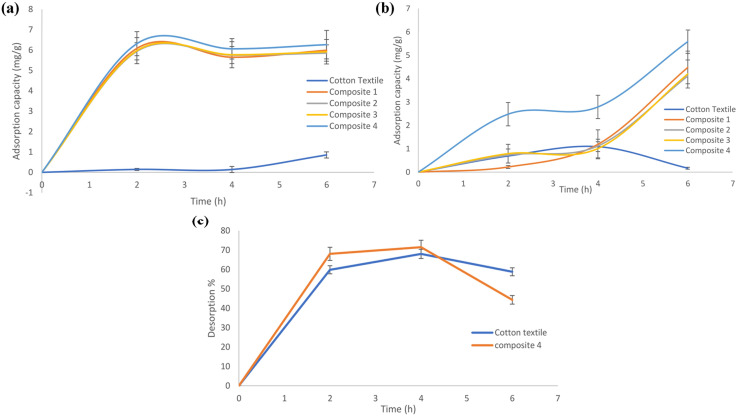
Cotton and cotton-CS composites adsorption capacity in (a) PbO solution; (b) Pb(NO_3_)_2_ solution, both at pH 5; and (c) desorption % of lead ions in HNO_3_ solution. Published under the CC BY license^154^ Copyright 2021 The authors, Published by MDPI, Basel, Switzerland.

In Fig. 13(a and b), the amount of Pb(ii) adsorbed is presented. After the first 2 hours of contact, the PbO solution adsorbed Pb^2+^ at a rate of 6 mg g^−1^ of composite, which is nearly seven times higher than with cotton. Conversely, with the Pb(NO_3_)_2_ solution, composite adsorbed 2 mg g^−1^ after 6 hours, about five times more than cotton, with composite 4 adsorbing the maximum concentration of Pb^2+^. The Pb^2+^ of the complexes generated between the cotton-CS composite and lead forms a bond with the NO_3_^−^ and is rinsed out of the cotton-CS composites, desorbing nearly 71.5% from the composite after two hours. The desorption determination was performed using HNO_3_ solution (Fig. 13(c).^154^

### 5.5. Drug delivery application

Lastly, the biomedical application of CS-coated fabrics plays an essential role in transdermal and localized drug delivery (see Table 4). The capability of polymers to encapsulate therapeutic agents and release them in a controlled manner opens new avenues in wound dressings and healthcare apparel. For instance, it was reported that cellulose fabrics treated with CNPs loaded with antibiotics like ciprofloxacin and tetracycline showed high antimicrobial efficacy while maintaining biocompatibility.^155^ Salama *et al.* (2022) demonstrated strong antimicrobial activity and reported that it increased the surface area, thereby improving biological activity to develop a nanotechnology-based controlled drug delivery device. These applications extend CS role from a finishing agent to an active medical facilitator.^156^

**Table 4:** Application of CS coated textile in the drug delivery and wound dressing field

Application area	Textile type	Additional agent	Application	Valuable data	*In vivo*/*in vitro*	Ref.
Drug delivery	Viscose cellulose fibers	CNPs/sodium tripolyphosphate	Antimicrobial agents themselves or as drug reservoirs	Polydispersity index of 0.168 and 0.175, encapsulation efficiency 87%, tested against *S. aureus*, *E. coli*, and *C. albicans*		157
	Cellulose-based cotton and viscose	CNPs loaded with aloe vera extract	Nanotechnology-based controlled drug delivery devices	↑Surface area, ↑biological activity, bacterial reduction 98.01 against *S. aureus*, and 99.98 against *E. coli*, viability (74–79) %	*In vitro* Hep. G2 cells	156
	100% cotton	CS and Si softener	Antibacterial and anti-inflammatory drug delivery	↓AP 322 m^3^ h^−1^ m^2^, *E. coli* and & *S. aureus*, ↓fabric stiffness, ↓fabric bending length, ↑washing durability	*In vitro*	158
Wound dressing	Non-woven CS	CS/hyaluronan		↑Swelling percentage, *E. coli*, *S. aureus* reduction rate ↑hydrophobicity, wound closure was 60% after 14 days	*In vitro* NIH-3T3 and HaCaT	159
	Tencel/cotton non-woven	CS/chlorhexidine	Develop an antibacterial textile covered by LBL coating	Cytocompatibility of polyethylene terephthalate, 99.0–104.4%, ↓cell vitality, ↑chlorhexidine adsorbed CHX from 45.1 mg g^−1^ (PEM11) to 143.7 mg g^−1^, against *S. aureus* and *E. coli*	*In vitro*, epithelial cell line (L132)	54
	Cotton knitted (CF: 150 g m^−2^)	CS/carboxymethylated	Effective hemostatic material in trauma treatment	↑Swelling ability, ↓crystallinity, ↑blood accumulation, ↑blood clotting	*In vitro*, CHI-MCF-0.39	160

**Fig. 14:**
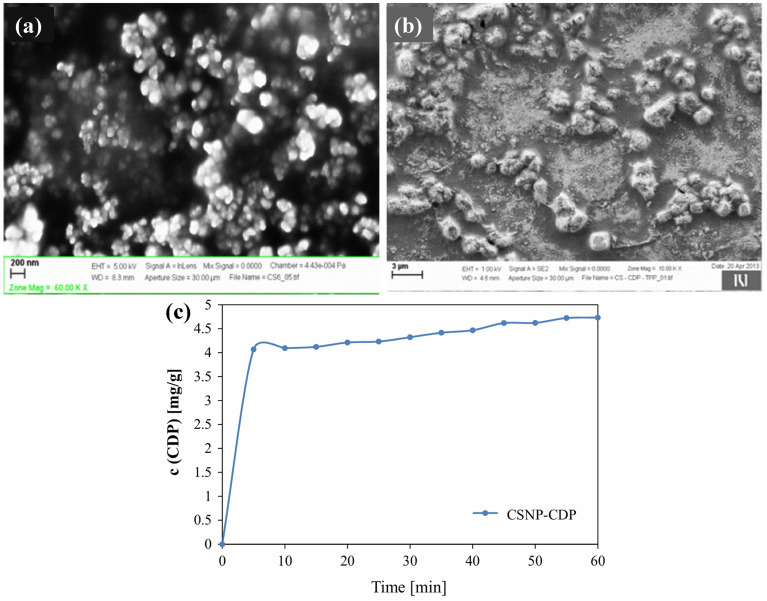
SEM images of drug-loaded nanoparticles on viscose fibers: (a) CNPs, (b) clindamycin 2-phosphate (CDP)-loaded CNPs, and (c) drug release from CDP-loaded particles attached to fibers at pH 7. Reproduced with permission^157^ Copyright 2016, Springer Science + Business Media Dordrecht.

According to Ristić (2017), CNPs with cellulose fiber used as drug delivery system. In Fig. 14 presents the morphology of drug-loaded nanoparticles on viscose fibers, showing SEM images of (a) CNPs, (b) CDP-loaded CNPs. Semispherical particles measuring 1 µm are shown in the SEM image of CDP-loaded CNPs (Fig. 14(b)), whereas plain CNPs (Fig. 14(a)) displayed sizes ranging from 50 to 250 nm. Drug adsorption on the particle surface was shown by the CDP-loaded CNPs uneven surface in comparison to the CNPs.^157^ Drug release tests were conducted in MQ water (pH 7) at 37C to stimulate body temp and a higher pH level, which is common of vaginal infections that require medication. Using a UV spectrophotometer, the concentration of the released medication in solution was ascertained. Fig. 14(c) displays the drug release profile of CDP-loaded particles bonded to fibers. After 5 minutes, the majority (around 86%) of the CDP had already been released a phenomenon known as the burst release effect and after 1 h, a maximum value of 4.7 mg CDP per 1 g of fibers was noted. Because hydrophilic medicines dissolve readily, burst release is common.^157^

### 5.6. Wound dressing application

CS-coated textiles are exhibiting significant attention in the biomedical sector, in the field of wound dressings (see Table 4). Using CS-based textile in wound dressing, which increases patient comfort and reduces the pain in the wound area.^160^ Non-woven fabrics coated with CS loaded hyaluronan showed more than 90% inhibition in *S. aureus* and *E. coli*, and achieved 60% wound closure after 14 days in the *in vitro* studied.^159^ Similarly,^160^ reported the increased blood clotting and swelling ability using carboxymethylated CS-treated fabrics. These fabrics support faster healing and lower infection risk, making them ideal for surgical gauze and trauma treatment.^160^

**Fig. 15:**
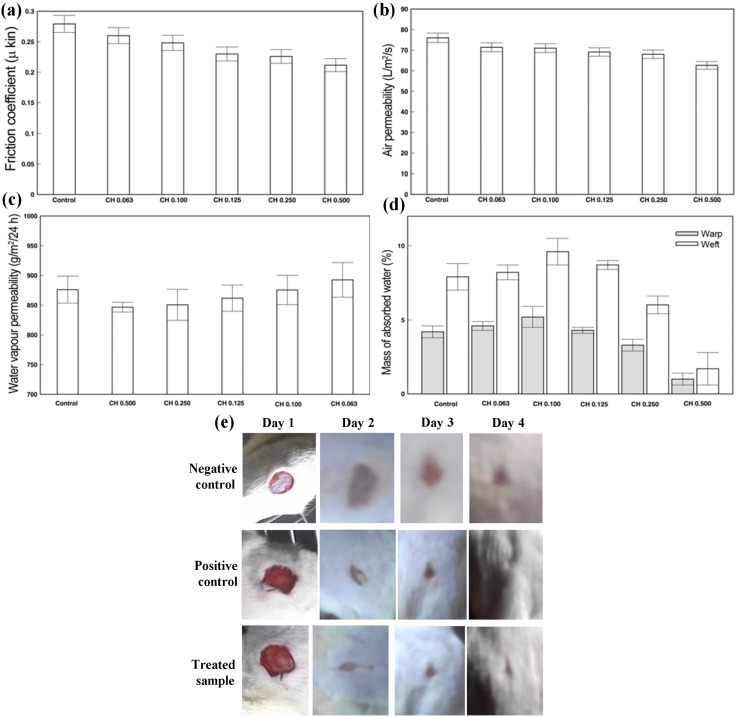
Ideal wound dressing properties of CS coated gauzes (a) coefficient of kinetic friction; (b) air permeability; (c) water vapor permeability; (d) mass of absorbed water (%) in warp (grey) and weft (white) directions; reproduced with permission^89^ Copyright 2019, Springer Nature and (e) photographic images of rats with burn wounds treated with cotton gauze (negative control), treated with Dermazin Ointment (positive control) and treated with cationized cotton gauze fabrics/AgNPs/drug/CS/salicylaldehyde (SA) fabrics taken on 0, 4, 8 and 12 days. Reproduced with permission^59^ Copyright 2020, Elsevier B.V.

Souza *et al.* (2020) shows the thermo-physiological properties of surgical cotton gauze coated with CS and its effectiveness for the prevention of bacterial colonization. In Fig. 15(a) it is clear that the coefficient of kinetic friction decreases with the increase in CS concentration. The result showed a decrease of 24% in the cotton gauze coated with 0.5 wt% of CS in relation to control gauze. CS appears to dramatically lower friction, which may prevent sheet burns, upper dermal skin layers, and epidermal damage. In Fig. 15(b), represents the values of air permeability of the gauzes coated with different concentrations of CS. When the concentration of CS increases, air permeability decreases, indicating significant changes in the porosity of the cotton gauze following CS addition. The highest air permeability is found in control gauze, which is unable to keep the wound environment reasonably moist. The inter-fiber distance and number of channels decrease as a result of CS coating, which partially fills the voids between warp and weft directions. Only the concentrations of CS 0.125, 0.250, and 0.5 wt% have adequate air permeability to guarantee and maintain ideal wound healing conditions, according to presented data.^89^ It is evident in Fig. 15(c) that the addition of CS causes a modest decrease in water vapor permeability; nevertheless, a noticeable effect requires a concentration of at least 0.125 wt% of CS. Water uptake in the weft direction is double that in the warp direction, as seen in Fig. 15(d). The fabric coated with 0.5 wt% CS exhibits a remarkable reduction in water uptake of almost 77% in the warp direction and 78% in the weft direction. These findings demonstrated that adjusting the CS concentration of the gauzes might modify their water absorption capacity and, in turn, their capability to remove exudate from the wound.^89^

Data presented in Fig. 15(e) demonstrate significant improvements in burn wound healing in treated sample (cationized cotton gauze fabrics/AgNPs/drug/CS/SA) compared with the negative control. The positive control group had an average wound contraction of 96.2%, which was the maximum healing efficiency. Although treated sample exhibited statistically significant better contraction value of 91.2% (compared with only 32% in the negative control group). The biopolymer Schiff base's capacity to regulate inflammatory cell activity and encourage the creation of granulation tissue may be the cause of this improved healing performance. Furthermore, the depolymerization of CS that releases *N*-acetyl-d-glucosamine may promote collagen deposition and fibroblast proliferation, improving the results of wound healing.^59,161,162^

Table 4 outlines the application of CS-coated textiles in the drug delivery and wound dressing field. In drug delivery applications, when CS is functionalized with textiles (cotton, viscose), their drug dispersibility, stability, efficacy, surface area, biological activity, and washing durability increase, while toxicity, fabric stiffness, *etc.* decrease. For example, when CNPs loaded with *Aloe vera* extract in cotton and viscose fabrics, their surface area, biological activity, and bacterial reduction decreased in Hep. G2 cells.^156^ In addition, CS is modified with textiles used in wound healing applications. When knitted cotton is functionalized with CS/carboxymethylated, their swelling ability, blood accumulation, and blood clotting increased, and crystallinity decreased in CHI-MCF-0.39 cell, which is effectively used as a hemostatic material in trauma treatment.^160^

## 6. Challenges

In large-scale production and use, the most vital problem is solubility. Though CS functionalization has been used for increasing its solubility.^163^ But CS is not soluble without acidic aqueous media (when pH is below 6.5), and that severely limits its compatibility with neutral processing conditions.^164^ Moreover, due to the limitations of CS fibers, which include higher electrostatic, inefficient mechanical strength, and elevated cost, CS fibers are commonly utilized as an assistant to secondary fibers in this industry. This can increase the commercial utility of the CS fibers and get the best output of CS fibers by keeping up the scientifically proven characteristics of CS in the textile. Besides, 100% CS also has drawbacks, such as being unwastable, having not that good softness and elasticity, and being too thin to be fixed in place.^165^ PES textile as a CS carrier should be surface altered for designing a stable bioactive fabric structure of CS and PES.^166^ One of the major drawbacks of CS-treated textiles is their poor wash durability.

The hydrophobic nature of polymer materials prevents the deposited coating from adhering properly or dispersing uniformly across the polymer surface. Polymer surfaces must first be activated with specific functional groups for CS coatings to effectively adhere to. CS is composed of a network of polysaccharides with amine-NH_2_ groups. In an acidic environment, these groups can be protonated to produce NH^3+^. The positively charged amine groups can electrostatically bind negatively charged molecules. Many synthetic polymers used in biomedicine lack carboxyl groups and other negatively charged surface functional groups. Consequently, CS will not adhere well to the surface of these polymers.^167^ For improving the CS stability and mechanical properties, chemical modifications are needed, many of which show cytotoxicity.^168^ There is a gap in research on the synthesis of CS from the most cost-friendly biological sources and its conversion into nanoscale materials. To increase production, CS formation remains challenging due to cost-intensive media and limited yields.^169^ The degree of deacetylation is expensive for CS. Hence, the production cost is high.^170,171^

## 7. Future prospect

Natural cross-linkers offer promising routes for sustainably functionalizing CS. For example, Genipin is used for its cross-linking ability. Cross-linking improves stability and structural integrity. Improving the stability and integrity of CS would increase their mechanical strength and would also retard their degradation percentage, and this would enhance their application in wound dressing, and tissue engineering.^171^ Moreover, ionic liquids also offer routes for functionalizing CS.^172^ Usage of CS as a material for developing smart textiles is increasing due to its biocompatibility, biodegradability, film-forming ability, and chemical reactivity.^20^ Research has developed an injectable, cross-linked, thermo-responsive hydrogel made from CS that can be designed to disulfiram to prevent cancer cells, and these gel formation systems exhibit increased biocompatibility and cytotoxicity. The bio-altered fabric formulation, which involves applying stimuli-responsive hydrogel-based drug delivery systems (developed by CS) on fabrics, was reported to be capable of delivering both drugs and moisture to the damaged areas of eczema (atopic dermatitis).^173^

CS films are surveyed as vital tools for application in regenerative medicine. CS nanocomposites are used as promising tools in regenerative medicine. CS films can accelerate wound healing by improving repair and inflammatory cells. Moreover, PVA-CS scaffolds support versatile tissue engineering. Preclinical studies exhibit that the PVA-CS film can be fabricated and altered to fit the specific uses of individual tissues and organs. PVA-CS can be mixed with other materials and technologies to increase its regenerative ability.^174^ Organic impurities such as parabens, antibiotics, dyes, microplastics, pesticides, PAHs, and PCBs are among the top groups of contaminants that constitute a significant threat to the availability of clean water. There is a need for functional materials with good adsorptive characteristics that are also renewable, widely available, and sustainable. Among these materials are CS-based materials that are physically and chemically modified to improve absorption efficiency.^175^

Biodegradability property of CS-metal oxide films (ZnO, TiO_2_, and Fe_2_O_3_) and CS-altered graphene films (CS–GO–Ag) in a soil environment. These films have excellent mechanical properties and exhibit antibacterial activity. The biodegradability of CS, CS: Zn, and CS: Fe_2_O_3_ films was crucially proven by the ability of soil microorganisms to use CS films as a source of carbon and nitrogen. Films of CS-metal oxide exhibit soil biodegradability. CS has also gained significant attention for its versatile applications in agriculture, its advantageous environmental impact, its ability to boost plant immunity and improve soil health, and its lack of hazardous residues.

## 8. Conclusions

The continued evolution of CS-functionalized textile biomaterials marks a vital step forward in the intersection of bio-based polymers and textile engineering. As this review has outlined, CS offers a unique balance of desirable traits: natural origin, antimicrobial efficacy, biocompatibility, and chemical versatility, positioning it as an ideal candidate for both high-performance and sustainable textile applications. The modification and functionalization strategies explored, including crosslinking, grafting, and nanostructure incorporation, have enabled textiles to perform beyond their traditional roles. These enhancements support use cases in biomedical fields such as antimicrobial, wound healing, and drug delivery, as well as in broader industrial sectors such as filtration and protective clothing. Despite this promising outlook, several barriers remain, such as limited long-term durability under repeated washing, inconsistencies in wide production, and regulatory complexities hinder widespread adoption. Furthermore, the environmental footprint of chemical crosslinkers and the stability of bioactive functionalities over time demand ongoing attention. Bridging these gaps will require interdisciplinary collaboration among textile engineers, materials scientists, chemists, and industrial stakeholders. Looking ahead, the integration of CS-functionalized textiles with smart technologies such as sensors, responsive coatings, or wearable electronics could unlock even greater potential, especially in personalized medicine and adaptive industrial wear. Overall, CS-functionalized textiles represent a versatile and sustainable platform with strong potential for next-generation functional textiles, bridging the gap between environmental responsibility and high-performance material design.

## Author contributions

Md. Hanif Munshi contributed to the conceptualization, investigation, data curation, formal analysis, and writing of the original draft. Md. Fahmid Islam contributed to the formal analysis, and writing of the original draft. Md. Hafizul Islam and Nikhilesh Bhowmick carried out data curation, formal analysis, and visualization. Tauqir Khan contributed to methodology, data curation, validation, and validation. Md. Kamruzzaman contributed to methodology, supervision, data curation, validation, and editing of the manuscript. Tarikul Islam contributed to funding acquisition, formal analysis, supervision, validation, project administration, and critical review and editing of the manuscript. All authors discussed the results and approved the final manuscript.

## Conflicts of interest

The authors declared no conflict of interest.

## Data Availability

No primary research results, software or code have been included and no new data were generated or analysed as part of this review.
